# Functional excitatory to inhibitory synaptic imbalance and loss of cognitive performance in people with Alzheimer’s disease neuropathologic change

**DOI:** 10.1007/s00401-022-02526-0

**Published:** 2022-12-20

**Authors:** Pietro Scaduto, Julie C. Lauterborn, Conor D. Cox, Anna Fracassi, Tommaso Zeppillo, Berenice A. Gutierrez, C. Dirk Keene, Paul K. Crane, Shubhabrata Mukherjee, William K. Russell, Giulio Taglialatela, Agenor Limon

**Affiliations:** 1 Department of Neurology, Mitchell Center for Neurodegenerative Diseases, University of Texas Medical Branch at Galveston, Galveston, TX USA; 2 Department of Anatomy and Neurobiology, University of California Irvine, Irvine, CA USA; 3 Department of Laboratory Medicine and Pathology, University of Washington School of Medicine, Seattle, WA USA; 4 Department of Medicine, University of Washington, Seattle, WA USA; 5 Department of Biochemistry and Molecular Biology, University of Texas Medical Branch at Galveston, Galveston, USA

## Abstract

Individuals at distinct stages of Alzheimer’s disease (AD) show abnormal electroencephalographic activity, which has been linked to network hyperexcitability and cognitive decline. However, whether pro-excitatory changes at the synaptic level are observed in brain areas affected early in AD, and if they are emergent in MCI, is not clearly known. Equally important, it is not known whether global synaptic E/I imbalances correlate with the severity of cognitive impairment in the continuum of AD. Measuring the amplitude of ion currents of human excitatory and inhibitory synaptic receptors microtransplanted from the hippocampus and temporal cortex of cognitively normal, mildly cognitively impaired and AD individuals into surrogate cells, we found regional differences in pro-excitatory shifts of the excitatory to inhibitory (E/I) current ratio that correlates positively with toxic proteins and degree of pathology, and impinges negatively on cognitive performance scores. Using these data with electrophysiologically anchored analysis of the synapto-proteome in the same individuals, we identified a group of proteins sustaining synaptic function and those related to synaptic toxicity. We also found an uncoupling between the function and expression of proteins for GABAergic signaling in the temporal cortex underlying larger E/I and worse cognitive performance. Further analysis of transcriptomic and in situ hybridization datasets from an independent cohort across the continuum of AD confirm regional differences in pro-excitatory shifts of the E/I balance that correlate negatively with the most recent calibrated composite scores for memory, executive function, language and visuospatial abilities, as well as overall cognitive performance. These findings indicate that early shifts of E/I balance may contribute to loss of cognitive capabilities in the continuum of AD clinical syndrome.

**Supplementary Information:**

The online version contains supplementary material available at 10.1007/s00401-022-02526-0.

## Introduction

Alzheimer’s disease (AD) is the most common cause of dementia worldwide [104]. From the clinical to the molecular level, AD is characterized by progressive impairment of cognitive performance, brain atrophy, neuronal and synaptic loss, and pathologic aggregation of amyloid beta (Aβ) and hyperphosphorylated tau (pTau) proteins. Notably, prodromal AD is characterized by mild cognitive impairment (MCI) and a greater prevalence of seizures and abnormal electroencephalographic activity [14, 34, 62, 64, 65, 76]. Clinical and experimental AD animal model studies suggest that hyperexcitability and cognitive impairment may be mechanistically linked through synaptic abnormalities that disturb the excitatory to inhibitory (E/I) balance in circuits vulnerable to AD pathology [7, 13, 28, 59, 64, 65, 76, 88]. However, the balance between synaptic excitatory and inhibitory inputs onto cortical neurons is maintained at a near constant level by tight homeostatic mechanisms, despite fluctuating activity [94]. Small changes in the E/I ratio can have large effects on neuronal activity that control spike timing and network rhythms and implement functional brain states [4, 13, 22, 38, 52, 89, 99], and since the production of Aβ and pTau oligomers depends on neuronal activity, hyperexcitability may accelerate the transition to dementia [16, 95]. Recently, using near-simultaneous electrophysiological recordings of human synaptic AMPA receptors (AMPARs) and GABA_A_ receptors (GABA_A_Rs) and unbiased counting of excitatory and inhibitory postsynaptic densities by fluorescence deconvolution tomography, we found functional and anatomical pro-excitatory shifts of the global synaptic E/I ratio in the AD parietal cortex (PCx) [44], an area known to be hyperactive in people with AD [49, 101]. However, whether pro-excitatory changes are also observed in brain areas affected early in AD, and if they are emergent in MCI, has not been established. Most important, whether global synaptic E/I imbalances correlate with the severity of cognitive impairment in the continuum of AD clinical syndrome is currently unknown.

In this study, we determined the E/I ratios in the postmortem medial human temporal cortex (TCx) and hippocampus from donors with MCI or AD dementia and compared them with control donors with normal cognition (CTRL) using orthogonal approaches. The TCx and hippocampus exhibit pTau and Aβ pathology in AD, and thus they are among the most studied brain areas and structures in AD research. Moreover, abnormal early high activation of the hippocampus correlates with the decline of cognitive performance in people at risk of AD [63], and individuals affected by AD pathology and subclinical epileptiform activity have faster cognitive decline than those who did not present such abnormalities [91].

Using a multilevel analytical approach to assess TCx and hippocampus from a cohort with a wide range level of pathology and cognitive impairment, we show convergent evidence of broad cortical pro-excitatory changes, driven by reduction of inhibitory neuronal markers, disorganization of GABAergic synapses and increase in AMPARs function, that strongly correlate with overall and discrete metrics of cognitive performance in AD.

## Materials and methods

### Human tissue

The University of California Irvine (UCI) Institute for Memory Impairments and Neurological Disorders through the Alzheimer's Disease Research Center (UCI-ADRC) provided hippocampi and medial temporal cortices from CTRL, MCI and AD donors as well as their last Mini-Mental State Examination (MMSE) score before death (Supplementary dataset 1, online resource). There was no effect of the time elapsed between the last cognitive examination and death with the MMSE score; Spearman’s correlation rho = − 0.155; *p* = 0.42. For the TCx, tissue blocks, that included a well-defined sulcus with portions of gyri present in both sides, were dissected from Brodmann area 21. For hippocampus, multiple thin coronal slices from the hippocampus were provided by the UCI brain bank. All cases were de-identified and coded by the tissue bank. For the present studies, the samples were recoded and processed for all analyses with the experimenter blinded to the donor and group. The study was reviewed by the Institutional Review Board of the University of Texas Medical Branch and categorized as a non-human subject study. All brains were processed within a post-mortem interval of 6.17 h. Further clinical and demographic data, such as sex, ethnicity, *APOE* genotype and AD neuropathologic change, are available in Supplementary dataset 1, online resource.

### Synaptosome fraction preparation

Synaptosome fractions were obtained from freshly frozen hippocampi (8 non-demented CTRL, 8 MCI and 11 AD) and medial TCx (6 CTRL, 6 MCI, and 6 AD). The synaptosome-enriched fraction was prepared using the Syn-PER reagent protocol (Thermo Fisher Scientific—Waltham, MA, USA) with proteinase inhibitors (Thermo Fisher Scientific—Waltham, MA, USA) to reduce proteolysis and denaturation, following the manufacturer’s instructions [75]. We chose the commercially available Syn-PER method to reduce internal variation and to facilitate the comparison with results from other research groups using the same approach. This method preserved a high number of synaptosomes in quantities comparable to other synaptosome isolation methods [29], while preserving the function of AMPA and GABA_A_ receptors in our microtransplantation of synaptic membranes experiments [44]. Briefly, brain tissue from the TCx (single 20 µm slice) and hippocampus (specimens’ weight average CTRL 36 ± 3.8 mg, MCI 40 ± 3.5 mg, and AD 39 ± 3.0 mg) from each individual was homogenized using polytetrafluoroethylene and glass tissue grinders. After two rounds of centrifugation (1200*g* × 10 min and 15,000*g* × 20 min at 4 °C), we obtained three fractions, S1 (soluble cytosolic elements), P1 (nuclei, myelin, and large nonhomogenized tissue) and P2 (synaptosome enriched fraction), from each preparation. These fractions were stored at − 80 °C. A DeNovix QFX fluorometer instrument (DeNovix Inc. Wilmington, USA) and Qubit reagents (Invitrogen—Thermo Fisher Scientific—Waltham, MA, USA) were used for protein quantification. Supplementary Fig. 1a (online resource) shows Electron Microscopy (EM) images with intact synaptosomes from pooled P2 preparations, pooling was necessary to preserve sample for functional and proteomics studies. Supplementary Fig. 1b (online resource) shows strong presynaptic and postsynaptic enrichment of proteins in P2 samples, measured by label-free proteomics (see Proteomics analysis section), whereas that of non-synaptic components was not statistically significant using the SynGO database [41] (Supplementary dataset 2, online resource).

### P2 immunostaining and flow cytometry

Because of the limited amount of human synaptic fraction, we pooled each subject in CTRL, MCI, and AD groups. Protein concentration in each donor’s P2 fraction was adjusted to 2 mg/mL with Syn-PER, then identical volumes from each P2 were combined in P2 fraction pools for each group. These P2 fraction pools were removed from a − 80 °C freezer and gradually thawed in ice for 20 min. Then 1 µL of the P2 fraction was transferred into a new Eppendorf tube containing 200 µL of 4% paraformaldehyde, pH 7.4, and incubated for 1 h at 4 °C. Within the incubation time, after 30 min, the samples were gently mixed by pipetting up and down 3 times and placed back at 4 °C for the remaining 30 min. PBS (400 μL) (Thermo Fisher Scientific—Waltham, MA, USA, cat#TA125PB) was added, and the samples were centrifuged for 8 min at 5000*g* to pellet the P2 fraction. The P2 fraction was resuspended in 150 μl permeabilization buffer (PBS + Tween 20 0.3%), incubated for 20 min at 32 °C and centrifuged for 8 min at 5000*g*. The supernatant was discarded, and the pellet was resuspended in 40 µL of blocking solution (PBS + FBS 2%) and incubated with post-synaptic antibody overnight. Anti-PSD95 (1:80, Novus-NB300-556AF647) or Anti-GPHN (1:100, Abcam-Ab32206) antibodies were used to label postsynaptic densities. GPHN required an additional 1 h incubation with secondary antibody (1:400, Invitrogen-A11008). After washing with PBS to eliminate nonspecific binding, flow cytometry analysis was performed on P2 fractions using Guava 3.3 software and a Guava EasyCyte flow cytometer (EMD Millipore). A size-gate based on forward and side scatters was built using size beads (Spherotech Inc.). P2 samples were diluted 1:500 in PBS and then loaded into the flow cytometer. The instrument was set to count 5000 events within the main gate. Each particle within the synaptosome gate was assigned a value of fluorescence intensity: red (PSD95) or green (GPHN) fluorescence. Statistical comparisons to identify the group effects used a one-way ANOVA followed by Tukey HSD and Dunnett’s multiple comparisons tests. In all cases, *p* < 0.05 was considered significant.

### Oocyte extraction and isolation

Oocytes from *Xenopus laevis* were used to perform MSM as previously reported [44, 97]*.* Frogs were anesthetized in a bath containing 0.17% tricaine for 10–15 min before extracting the ovaries, following procedures in accordance with the National Institutes of Health Guide for the Care and Use of Laboratory Animals at the University of Texas Medical Branch at Galveston (IACUC: 1803024). To remove the follicular layer, oocytes were incubated for 4.5 h at 31 °C with 0.2% collagenase type I in Barth’s solution [88 mM NaCl, 1 mM KCl, 0.41 mM CaCl_2_, 0.82 mM MgSO_4_, 2.4 mM NaHCO_3_, 5 mM HEPES (pH 7.4)]. Finally, the oocytes were washed using Barth’s solution. Healthy spherical oocytes with no signs of tear and developmental stages V–VI were selected for MSM experiments.

### Microtransplantation of synaptic membranes

Microtransplantation of synaptic membranes (MSM) is an extension of the microtransplantation of membranes method that has been used in the academy and in the pharmaceutical industry to obtain previously inaccessible information about the function of native receptor complexes in many human disorders (for review of the microtransplantation of membranes method please see [21, 103]). Because MSM provides functional information directly from native receptors, it has been also used to validate synaptoproteome differences of synaptic receptors across cortical Brodmann areas in the human neocortex [73]. Here we performed MSM as previously described [44, 53, 97]. Briefly, defolliculated oocytes (stage V–VI, diameter 1–1.5 mm) were injected with 50 nL of P2 fraction at 2 mg/mL, using nanoject 2 auto-nanoliter injector (Drummond Scientific Company, Broomall, PA, USA) mounted with a borosilicate glass micropipet (O.D. 2 mm, I.D.:1.16 mm). After injection, microtransplanted oocytes were incubated in Barth’s solution at 18 °C. A two-electrode voltage-clamp (TEVC) was executed into the oocytes 24–32 h post-injection. Microelectrodes for TEVC were filled with 3 M KCl, and their resistance ranged from 0.5 to 3.0 MΩ. Microtransplanted oocytes were pierced and recorded into a small, customized chamber (volume 70 µL) continuously perfused (≈ 5 ml/min) with Ringer’s solution (115 mM NaCl, 2 mM KCl, 1.8 mM CaCl_2_, 5 mM HEPES [pH 7.4]) at room temperature (19–21 °C). Oocytes were clamped at − 80 mV using an Oocyte Clamp OC-725C amplifier. To record and store data, we used WinEDR version 3.2.7 and 3.9.1 Strathclyde Electrophysiology Software (John Dempster, Glasgow, UK). To filter currents, we used Kemo^®^ BenchMaster 8. To exclude any artifact and validate the success of receptors transplant, naïve and microtransplanted oocytes were perfused with receptor agonists to activate synaptic currents: GABA (Sigma-Aldrich—St. Louis, MO, USA) for GABA_A_R, and kainate (Tocris—Minneapolis, MN, USA) for AMPAR. As we previously showed, only microtransplanted oocytes produced an electrophysiological response when perfused with agonists [44]. We previously demonstrated that kainate activation of receptors other than the AMPAR current in MSM experiments is negligible [44]. All the drugs were dissolved into Ringer’s solution and perfused into the chamber in which the oocytes were clamped. Synaptic currents were measured at the peak, referred to as the maximum current. Drug perfusion was stopped after the current reached the plateau for kainate or the maximum peak before desensitization for GABA. For all measurements, for each donor, electrophysiological recordings were performed at least in triplicate (three oocytes) in batches of oocytes from two to four different frogs, balancing the groups for an equal number of donors in each experimental run. Statistical comparisons to identify the effect of diagnosis used the mean of each metric for each donor as an experimental unit in a one-way ANOVA, followed by post hoc Tukey’s test and Dunnett’s multiple comparisons versus control test (JMP, version 14). In all cases, *p* < 0.05 was considered significant. The Pearson product moment was used for all linear correlations using JMP version 14 (SAS Institute, Cary, NC, USA).

### Proteomic analysis

P2 fractions (2 µg/µL) were sonicated 6 times for 5 s as for MSM. Approximately 5 µg of sample was prepared as described [81]. Briefly, the agarose bead-bound purified proteins were washed several times with 50 mM TEAB, pH 7.1, before being solubilized with 40 µL of 5% SDS and 50 mM TEAB, pH 7.55, and incubated at room temperature for 30 min. The supernatant containing the proteins of interest was then transferred to a new tube and reduced by adding 10 mM TCEP solution (Thermo, #77720) and incubating at 65 °C for 10 min. The sample was then cooled to room temperature, and 3.75 µL of 1 M iodoacetamide acid was added and allowed to react for 20 min in the dark, after which 0.5 µL of 2 M DTT was added to quench the reaction. Five microliters of 12% phosphoric acid was added to 50 µL of protein solution. Then, 350 µL of binding buffer (90% methanol, 100 mM TEAB final; pH 7.1) was added to the solution. The resulting solution was added to an S-Trap spin column (protifi.com) and passed through the column using a bench top centrifuge (30 s spin at 4000*g*). The spin column was washed with 400 µL of binding buffer and centrifuged. This process was repeated three times. Trypsin was added to the protein mixture at a ratio of 1:25 in 50 mM TEAB, pH = 8, and incubated at 37|°C for 4 h. Peptides were eluted with 80 µL of 50 mM TEAB, followed by 80 µL of 0.2% formic acid, 80 µL of 50% acetonitrile, and 0.2% formic acid. The combined peptide solution was then dried in a speed vac and resuspended in 25 µL of 2% acetonitrile, 0.1% formic acid, and 97.9% water and placed in an autosampler vial. Then, 1 µL was analyzed by nanoflow liquid chromatography-tandem mass spectrometry (nanoLC-MS/MS) using a nano-LC chromatography system (UltiMate 3000 RSLCnano, Dionex, Thermo Fisher Scientific—Waltham, MA, USA). The nanoLC-MS/MS system was coupled online to a Thermo Orbitrap Fusion mass spectrometer (Thermo Fisher Scientific—Waltham, MA, USA) through a nanospray ion source (Thermo Fisher Scientific—Waltham, MA, USA). A trap and elute method was used to desalt and concentrate the sample while preserving the analytical column. The trap column (Thermo Fisher Scientific—Waltham, MA, USA) was a C18 PepMap100 (300 µm × 5 mm, 5 µm particle size), while the analytical column was an Acclaim PepMap 100 (7 µm × 25 cm) (Thermo Fisher Scientific—Waltham, MA, USA). After equilibrating the column in 98% solvent A (0.1% formic acid in water) and 2% solvent B (0.1% formic acid in acetonitrile [ACN]), the samples (2 µL in solvent A) were injected onto the trap column and subsequently eluted (300 nL/min) by gradient elution onto the C18 column as follows: isocratic at 2% B, 0–5 min; 2% to 28% B, 5–120 min; 28% to 40% B, 120–124 min; 40% to 90% B, 124–126 min; isocratic at 90% B, 126–130 min; 90% to 2%, 130–132 min; and isocratic at 2% B, until the 150 min mark. All LC–MS/MS data were acquired using XCalibur, version 2.4.0 (Thermo Fisher Scientific—Waltham, MA, USA) in positive ion mode using a top speed data-dependent acquisition (DDA) method with a 3 s cycle time. The survey scans (*m*/*z* 350–1500) were acquired in the Orbitrap at 120,000 resolution (at *m*/*z* = 400) in profile mode, with a maximum injection time of 100 ms and an AGC target of 400,000 ions. The S-lens RF level was set to 60. Isolation was performed in the quadrupole with a 1.6 Da isolation window, and CID MS/MS acquisition was performed in profile mode using a rapid scan rate with detection in the ion trap using the following settings: parent threshold = 5000; collision energy = 32%; maximum injection time 56 ms; AGC target 500,000 ions. Monoisotopic precursor selection (MIPS) and charge state filtering were on, with charge states 2–6 included. Dynamic exclusion was used to remove selected precursor ions, with a ± 10 ppm mass tolerance, for 15 s after acquisition of one MS/MS spectrum. Tandem mass spectra were extracted, and the charge state was deconvoluted using Proteome Discoverer (Thermo Fisher Scientific—Waltham, MA, USA, version 2.2.0388). Deisotoping was not performed. All MS/MS spectra were searched against a UniProt Homo sapiens protein FASTA database using Sequest. Searches were performed with a parent ion tolerance of 5 ppm and a fragment ion tolerance of 0.60 Da. Trypsin is specified as the enzyme, allowing for two missed cleavages. Fixed modification of carbamidomethyl (C) and variable modifications of oxidation (M) and deamidation were specified in Sequest. Control donors S4 and S27, and AD donor S19 did not have protein abundance enough to pass internal quality control from proteomics and could not be incorporated in the proteomic analysis. The Pearson product moment was used for all linear correlations using JMP version 16. Pro (SAS Institute, Cary, NC, USA).

### Gene ontology analysis

To determine enriched modules, a response screening analysis using linear regression and controlling for multiple comparisons was performed to find genes or proteins that linearly correlate with the variables used. Correlated genes and proteins with *p* < 0.001 were selected for GO analysis of cellular components. For synaptic proteomic analysis, we used the SynGO database using the initial conditions suggested by the website. For transcriptomic analysis, we used the whole transcriptome as background. A *p* value cutoff of 0.01, minimum enrichment of 1.5, and gene prioritization by evidence counting were determined using Metascape [100].

### Transcriptome analysis

To estimate the *t*E/I ratio in the hippocampus and TCx, publicly available RNA-seq data from the Aging, Dementia and Traumatic Brain Injury (ADTBI) study were analyzed [54]. The dataset was downloaded from the website (http://aging.brain-map.org/download/index). The first analysis was restricted to 20 donors that matched CTRL and AD parameters based on manual of mental disorders IV (DSM-IV) and CERAD (Consortium to Establish a Registry for Alzheimer's Disease) score. Eight cognitively intact donors (5 males, 3 females, 78–100 y/o with no dementia, CERAD = 0) and 12 donors with dementia and DSM-IV clinical diagnosis AD (6 males, 6 females, age-matched to controls) and severe neuropathologic change (CERAD = 3). The second analysis included all donors in the dataset. Fifty-six CTRL and 30 AD cases, excluding only demented individuals due to vascular, medical, multiple etiology or unknown causes (Supplementary dataset 3, online resource). The *t*E/I ratio for each donor was calculated based on the ratio of the fragments per kilobase per million mapped reads for the PSD95 transcript *DLG4* to *GPHN* mRNA. For significant differences in gene expression in CTRL versus AD donors, the two-tailed Student’s *t* test was used. The Pearson product moment was used for all linear correlations using JMP version 16 Pro.

### In situ hybridization analyses

Full-size high-resolution images of colorimetric ISH for vGluT1 (SLC17A7) and GAT1 (SLC6A1) mRNA-expressing cells in the TCx and hippocampus were downloaded from the ADTBI study (website—http://aging.brain-map.org/donors/summary). For both brain areas, the cases assessed were a subgroup of those used for the gene expression analyses because not all the cases had both RNA-seq and in situ hybridization (ISH) data; additionally, AD case H14.09.042 did not have TCx images, and AD case H14.09.098 did not have hippocampal images. For TCx, 7 CTRL and 7 AD cases were analyzed. For each case, the TCx vGluT1 and GAT1 images were cropped to the same size sample field (e.g., 9,000,000 µm^2^) to encompass all layers I–VI of the cortical field; the location of each sample field was matched between the images for each mRNA. Three cropped images for each mRNA were assessed per case, with the mean total area assessed for the CTRL and AD groups being 34,920,000 ± 2,545,480 µm^2^ and 34,191,429 ± 3,603,269 µm^2^, respectively (*p* = 0.669). Automated counts of labeled TCx cells were performed using scikit-image 0.16.2 and Python 3.7 as previously described [44]. Images were Gaussian blurred at 3-pixel sigma to remove small imaging artifacts and then thresholded using a Yen intensity threshold [96], which was found to be optimal across numerous thresholding methods blind-tested across several images. Cell objects were counted at multiple thresholds (10, 20, and 30 pixels), and while all yielded similar differences in E/I ratios between groups, the largest 30 µm^2^ size threshold size was chosen for final analyses. Values were expressed per 100,000 µm^2^.

For the hippocampus, which has well-defined anatomical subfields, 6 CTRL and 5 AD cases were selected for analyses using the criteria that each case had to have a visible dentate gyrus, region CA4 and CA3; this resulted in the exclusion of 1 CTRL and 3 AD cases with poorly defined regions. Since region CA1 was not present in all the final cases, this field was excluded from the analyses. Unlike the cortex, where excitatory and inhibitory cells are interspersed across the cortical layers, hippocampal excitatory cells are localized within a specific lamina (e.g., *stratum pyramidale* and, in humans, within the dentate hilar region referred to as CA4). Inhibitory cells are distributed throughout the hippocampus but also exclusively in the molecular layers that are devoid of excitatory neurons (e.g., CA3 apical dendritic field and the dentate gyrus molecular layer). Thus, for relative E/I ratios in the hippocampus, vGluT1 mRNA-positive cells were counted in sample fields of both the CA3 *stratum pyramidale* and CA4, and GAT1 mRNA-positive cells were counted in sample fields of the CA3 *stratum pyramidale*, CA3 apical dendritic field, and dentate gyrus molecular layer. For each mRNA, three cropped images were obtained per sample field for each case. The mean size of each sample field was as follows: CA3 *stratum pyramidale*, 450,000 µm^2^ (the location of the sample field was matched between the images for both mRNAs); CA4, 600,000 µm^2^; CA3 apical dendritic field, 320,000 µm^2^; dentate gyrus molecular layer, 500,000 µm^2^. Automated counts of GAT1-labeled cells were performed as described above, and objects were counted if they were larger than 30 pixels. Because the vGluT1-labeled cells in the hippocampal fields were more packed together and often touched other labeled cells, these cells were counted manually using the same size criteria to avoid potential confounds that would occur with automated counting. Images were imported into ImageJ, and cells were counted using the multipoint tool by three independent scorers blinded to cases and groups. The three scores for each image were then averaged for a final cell count for each vGluT1 image field. For final vGluT1 and GAT1 counts, numbers were averaged across all sample fields for each mRNA, and values of labeled cells were expressed per 100,000 µm^2^ (each region is shown singularly in Supplementary Fig. 10, online resource). For multivariate correlation analysis we used the total number of positive cells. Either a two-tailed unpaired Student’s *t* test or a two-tailed unpaired Mann–Whitney test was used for statistical analyses between the two groups and was conducted using Prism 9.1.1.

### Cognitive scores

The ADTBI cohort is a cohort originally designed to study TBI exposure with age-matched CTRL, given that the ADTBI cohort is a subset of brain donors from the larger Adult Changes in Thought (ACT) study, which is a community-based sample carried out by the University of Washington in the Seattle metropolitan area. To study associations different from TBI, the Allen Institute provides weights to reduce this selection bias. These weights were used in all analyses named weighed, as recommended in https://help.brain-map.org/download/attachments/9895983/Weighted_Analyses.pdf?version=1&modificationDate=1456179403835&api=v2. Weighted multivariate Pearson product moment was used for all linear correlations using JMP version 14. For linear regressions between the *t*E/I ratio and pathological markers with cognitive scores, a weighted forward stepwise regression using the minimum Bayesian information criterion as a stopping rule was used to select the variables and implement the model in JMP 15. Pro.

## Results

### Electrophysiological metrics of global synaptic function correlate with disease severity.

**Fig. 1:**
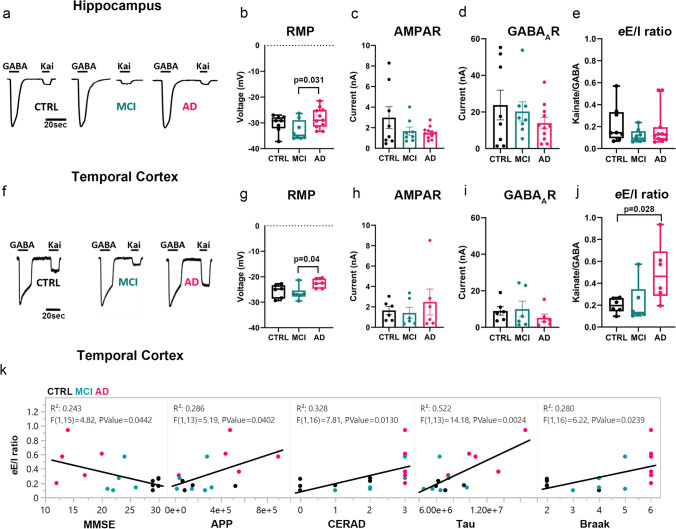
Differential perturbation of the *e*E/I balance in the hippocampus and TCx of AD donors.** a**, **f**, Representative electrophysiological responses from oocytes microtransplanted with human synaptic membranes from the hippocampus or TCx and perfused with 1 mM GABA or 100 µM kainate (Kai), an agonist of AMPARs. **b**, **g**, The resting membrane potential (RMP) of oocytes microtransplanted with human membranes was different across diagnostic groups in hippocampus (*F* (2, 24) = 3.71, *p* = 0.039) and TCx (*F* (2, 15) = 4.08, *p* = 0.038) and was more depolarized in AD compared to MCI (hippocampus post hoc Tukey *p* = 0.031; TCx post hoc Tukey *p* = 0.039). **c**, **h**, AMPAR responses to Kai were not significantly different across diagnostic groups in either the hippocampus (*F* (2, 24) = 1.78, *p* = 0.19) or the TCx (*F* (2, 15) = 0.46, *p* = 0.64). Each point is the average of the maximum response to Kai per donor. **d**, **i**, GABA_A_R responses to GABA did not show differences within diagnostic groups (hippocampus; *F* (2, 24) = 0.91, *p* = 0.42; TCx; *F* (2, 15) = 0.66, *p* = 0.53). **e, j,** Electrophysiological E/I balance (*e*E/I) was increased in the TCx (*F* (2, 15) = 4.58, *p* = 0.028 followed by Dunnett’s test *p* = 0.028 and Tukey’s test *p* = 0.039) but not in the hippocampus (*F* (2, 24) = 1.09, *p* = 0.35). The *e*E/I balance was calculated from the near-simultaneous recording of maximum responses of AMPARs and GABA_A_Rs in every single oocyte. Each point is the average of at least 3 oocytes per donor from 6 (hippocampus) or 5 (TCx) independent experiments (details in Supplementary dataset 4, online resource). In panels **c**, **d**, **h**, and **i**, bars are presented with standard error; in **b**, **g**, **e** and **j**, the box plots extend from the 25th to 75th percentiles, and the whiskers extend down to the minimum and up to the maximum value. One-way ANOVA was used in all tests. **k**, Pro-excitatory shift of the *e*E/I ratio in TCx is correlated with loss of cognitive performance, increase of APP abundance and CERAD, as well as with tau levels and Braak stage. Each dot represents the average value for each donor color coded as shown in **a**

**Fig. 2:**
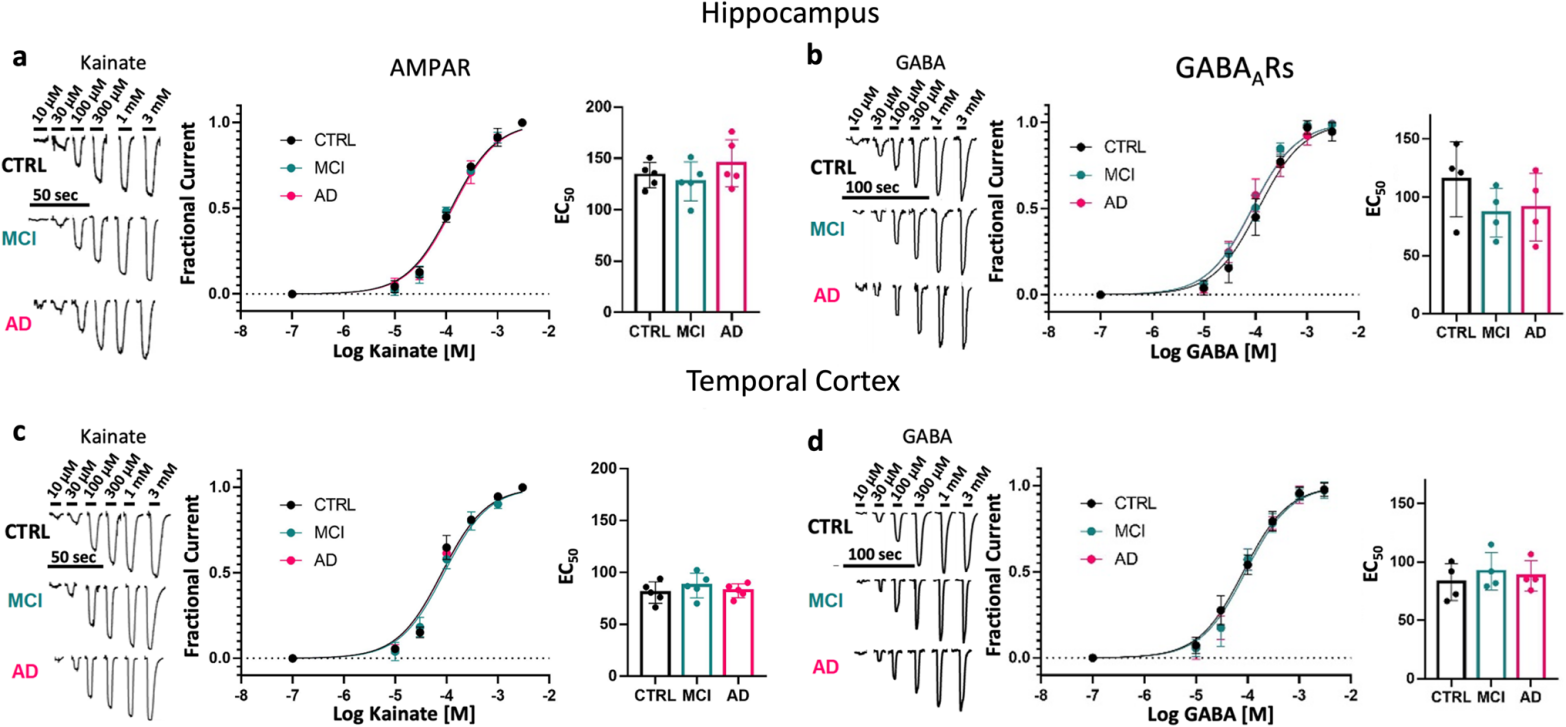
Preservation of synaptic receptor affinity. **a**, **c**, left, Representative recording of currents elicited by different concentrations of kainate on oocytes microtransplanted with pooled synaptosome fractions from the hippocampus and TCx of CTRL (hippocampus, *n* = 8; TCx, *n* = 6 donors), MCI (hippocampus, *n* = 8; TCx, *n* = 6 donors) and AD (hippocampus; *n* = 11; TCx *n* = 6 donors). **a**, **c**, Center, kainate-activating AMPAR concentration–current response relationships (hippocampus, *n* = 5 oocytes and TCx, *n* = 5 oocytes). **a**, **c**, Right, EC_50_ obtained from the AMPAR concentration–response curve did not show differences in the hippocampus (*F* (2, 12) = 1.16, *p* = 0.35) or TCx (*F* (2, 12) = 0.64, *p* = 0.55). **b**, **d**, Left, currents elicited by different concentrations of GABA on oocytes microtransplanted with the same pool of synaptosome preparations described above. **b**, **d**, Center, GABA-activating GABA_A_R concentration–current response relationships (hippocampus, *n* = 4 oocytes; TCx, *n* = 4 oocytes). **b**, **d**, Right, EC_50_ for GABA was not different in the hippocampus (*F* (2, 9) = 0.37, *p* = 0.70) or TCx (*F* (2, 9) = 1.22, *p* = 0.34). For all figures, the oocyte membrane potential was held at − 80 mV. Data were collected from two independent experiments, and all values were normalized to the maximum (see details in Supplementary dataset 5, online resource). Whiskers from each dose point represent standard deviations

To test the hypothesis that the human hippocampus and TCx in the continuum of AD are characterized by functional impairment of the synaptic E/I ratio, we first prepared samples enriched in synaptosomes (P2 fractions) from hippocampal tissue of 8 non-demented CTRL, 8 MCI and 11 AD donors, as well as medial TCx tissue from an additional 6 CTRL, 6 MCI and 6 AD donors provided by the UCI-ADRC (Supplementary dataset 1, online resource). Size-gated synaptosome-like particles were counted by flow cytometry using specific antibodies against the excitatory and inhibitory postsynaptic density proteins PSD95 and gephyrin, respectively. In hippocampus, PSD95-labeled particles showed trends of reduction in MCI and increasing in AD respect to controls (Supplementary Fig. 2, online resource). A significant difference was observed only between MCI and AD, suggesting that initial postsynaptic alterations present in MCI may be followed by compensatory changes in the opposite direction in AD. Interestingly, no differences were found in the total number of labeled and size-gated synaptosome-like particles of the hippocampus across diagnostic groups (Supplementary Fig. 2, online resource). However, total synaptosome-like particles were significantly reduced in the TCx of MCI and AD compared to CTRL (Supplementary Fig. 3, online resource), driven by a reduction of PSD95-labeled synapses, indicating an early cortical synaptic loss in people with AD-related cognitive impairment. Due to unequal amounts of synaptosome-like particles within groups, we prepared P2 aliquots that had the same protein concentration and used them for microtransplantation of synaptic membranes (MSM). MSM experiments were performed to determine the global electrophysiological synaptic E/I ratio (*e*E/I ratio). The *e*E/I ratio was defined as the maximum amplitude of AMPAR currents divided by the peak amplitude of the GABA_A_R current in the same microtransplanted cell (oocyte). The microtransplanted oocytes effectively inserted human native membranes containing functional synaptic AMPARs and GABA_A_Rs (Fig. 1a, f; Supplementary dataset 4, online resource). Oocytes microtransplanted with membranes from AD donors had a more depolarized resting membrane potential than those injected with membranes from MCI donors and a similar trend versus those injected with control membranes, possibly due to oligomeric Aβ pore-forming properties present in P2 fractions [10, 78] (Fig. 1b, g); indeed, the larger the abundance of the Aβ precursor protein (APP) measured in proteomics, the more positive the resting membrane potential of transplanted oocytes (*R*^2^ = 0.34; *p* = 0.023). No significant group differences in the maximum amplitude of ion currents elicited by activation of synaptic receptors were observed in either brain region (Fig. 1c, d, h, i). Notably, there was a modest correlation between the amplitude of currents elicited by microtransplanted receptors in the hippocampus and the score from the MMSE; the larger the amplitude of GABA_A_Rs currents, the better the cognitive performance score (*R*^2^ = 0.152; *p* = 0.049) (Supplementary Fig. 4a, online resource). A similar association was observed for AMPARs currents (*R*^2^ = 0.126; *p* = 0.075, Supplementary Fig. 4a, online resource). In the TCx, neither the amplitude of GABA_A_Rs nor AMPARs currents correlated with MMSE scores (Supplementary Fig. 4c, online resource), suggesting a potentially different mechanism underlying cortical dysfunction. Similar to our previous study of the PCx [44], the amplitudes of AMPARs and GABA_A_Rs currents from the hippocampus and TCx were highly correlated (*R*^2 ^= 0.751; *p* < 0.0001 for the hippocampus and *R*^2 ^= 0.356 for TCx; *p* < 0.0089), indicating strong regulation of the global E/I ratio (Supplementary Fig. 5, online resource). Notably, the slope of AMPARs vs GABA_A_Rs current in the TCx of AD donors was significantly higher compared to control and MCI donors (Supplementary Fig. 5, online resource). The strong correlation between AMPARs and GABA_A_Rs currents across all groups allowed us to calculate the *e*E/I ratio and evaluate its relationship with MMSE scores. The *e*E/I ratio was not different across diagnoses in the hippocampus (Fig. 1e) but was significantly shifted toward excitation in the TCx of AD donors (Fig. 1j). Notably, the *e*E/I ratio and MMSE scores were negatively correlated in TCx (*R*^2 ^= 0.243; *p* = 0.044, Fig. 1k, Supplementary Fig. 4a, online resource) but not in the hippocampus (*R*^2 ^= 0.028; *p* = 0.417, Supplementary Fig. 4c, online resource), and the greater the burden of neuritic plaques and neurofibrillary tangles (as per CERAD and Braak stages, respectively) in the TCx, the larger the *e*E/I ratio (Fig. 1k, Supplementary Fig. 4d, online resource). In the hippocampus, this association was not observed (Supplementary Fig. 4b, online resource). We further investigated whether the *e*E/I imbalance found in the TCx or lack thereof in the hippocampus was due to differences in the pharmacological affinity of synaptic receptors. Concentration–response curves for AMPARs and GABA_A_Rs currents did not show differences in their EC_50_ values across groups in the hippocampus (Fig. 2a, b) or TCx (Fig. 2c, d; Supplementary dataset 5, online resource), indicating that the alterations in the *e*E/I ratio in the TCx of donors with AD were not due to major changes in receptor affinity.

### Electrophysiological-anchored analysis of the synapto-proteome in TCx of donors with MCI and AD.

To investigate alternative causes for the increased *e*E/I ratio in the TCx of donors with AD, we evaluated the abundance of proteins in TCx synaptosome preparations using nanoflow liquid chromatography-tandem mass spectrometry (nanoLC–MS/MS). We found a total of 2902 proteins, of which 96% (2788 proteins; Supplementary dataset 6, online resource) were expressed by all groups. We also found that 421 proteins were differentially expressed between groups (*p* < 0.05) but only 27 of them passed False Discovery Rate, *p* < 0.05 (Supplementary dataset 7, online resource). Four of the five proteins that were increased in both MCI and AD respect to control form part of the LKB1 pathway known to promote tau phosphorylation via activation of MARK2 and PAR-1 [40, 92] (Supplementary Fig. 6; Supplementary dataset 8, online resource), while the rest of the proteins were reduced and involved in synaptic vesicle pathways and the metabolism of synaptic RNA, which has an important role controlling the composition of the local proteome. These changes indicate that early alterations by pathological processes impact the synaptic function in TCx of MCI and AD donors.

Because of the observed changes on the amplitude of the ion currents of microtransplanted receptors we evaluated the abundance of subunit proteins forming the pore of AMPARs and GABA_A_Rs in our preparations. We found expression of the GABA_A_R subunits α1, α3, α5, β1, β2, β3, and γ2 and the AMPAR subunits GluR1, GluR2, GluR3 and GluR4. Based on our previous findings of region-specific signatures of AMPAR and GABA_A_R subunits and their complementary relationships [77, 79], we analyzed whether the sum of all AMPAR subunits (ΣAMPARs) found in the proteomic analysis correlated with the sum of all GABA_A_R subunits (ΣGABA_A_Rs) and whether there were differences across groups. As expected from our electrophysiological results, ΣAMPARs and ΣGABA_A_Rs were also correlated (*R*^2^ = 0.59; *p* = 0.0008, Supplementary Fig. 7a, online resource); however, no differences in their abundance or ratio (Supplementary Fig. 7b, Supplementary dataset 19, online resource) were found between groups. We did not observe differences in the synaptic scaffolds PSD95 or gephyrin or their proteomic ratio (*p*E/I ratio: PSD95/gephyrin; Supplementary Fig. 7d, Supplementary dataset 19, online resource). The synaptic glutamate transporter vGLUT1 showed decreased levels in MCI subjects, but not in AD, compared to controls, whereas the synaptic GABA transporter GAT1 and the ratio vGLUT/GAT1 did not show differences (Supplementary Fig. 7c, Supplementary dataset 19, online resource). Importantly, the ion currents generated by activation of AMPAR (*e*AMPAR) followed the expected correlation with levels of ΣAMPAR subunits (*R*^2^ = 0.67; *p* = 0.0003), and with the levels of PSD95 (*R*^2^ = 0.313; *p* = 0.0301), but not with vGLUT1 (*R*^2^ = 0.166; *p* = 0.13) (Supplementary Fig. 7e, online resource). However, the amplitude of GABA_A_Rs currents did not correlate with ΣGABA_A_R subunits (*R*^2^ = 0.149; *p* = 0.155), GAT1 (*R*^2^ = 0.258; *p* = 0.0531), or gephyrin levels (*R*^2^ = 0.043; *p* = 0.46) (Supplementary Fig. 7f, online resource), suggesting that a loss of correlation between inhibitory markers and function of GABA_A_Rs contributes to the dissociation between *e*E/I ratio with *p*E/I, vGLUT/GAT1 and ΣAMPARs/ΣGABA_A_Rs ratios in the TCx (Supplementary Fig. 7g, online resource).

Because the electrophysiological function of AMPARs and GABA_A_Rs is strongly modulated by auxiliary and associated proteins, we further screened for proteins correlated (cutoff *p* < 0.05) with the amplitude of the currents elicited by these receptors [97]. We found 523 proteins positively correlated with *e*AMPARs, 256 with *e*GABA_A_Rs, and 142 with both (Supplementary dataset 9, online resource). Most of these proteins were found in gene ontology (GO) terms representing RAB geranylgeranylation, regulation of vesicle transport, energy control and modulation of chemical synapses (Supplementary Fig. 8a; Supplementary dataset 10, online resource). Repeating this analysis for each group confirmed most of the GO modules, but also revealed important differences between groups (Supplementary Fig. 8b, c, online resource). Specifically, there was minimal overlap between the proteins that correlated with AMPARs from those that correlated with GABA_A_Rs in the control group; however, this overlap increased in MCI and even more in AD, suggesting large synaptic remodeling and a potential loss of synaptic specialization as the pathology progresses (Supplementary dataset 11, online resource).

**Fig. 3:**
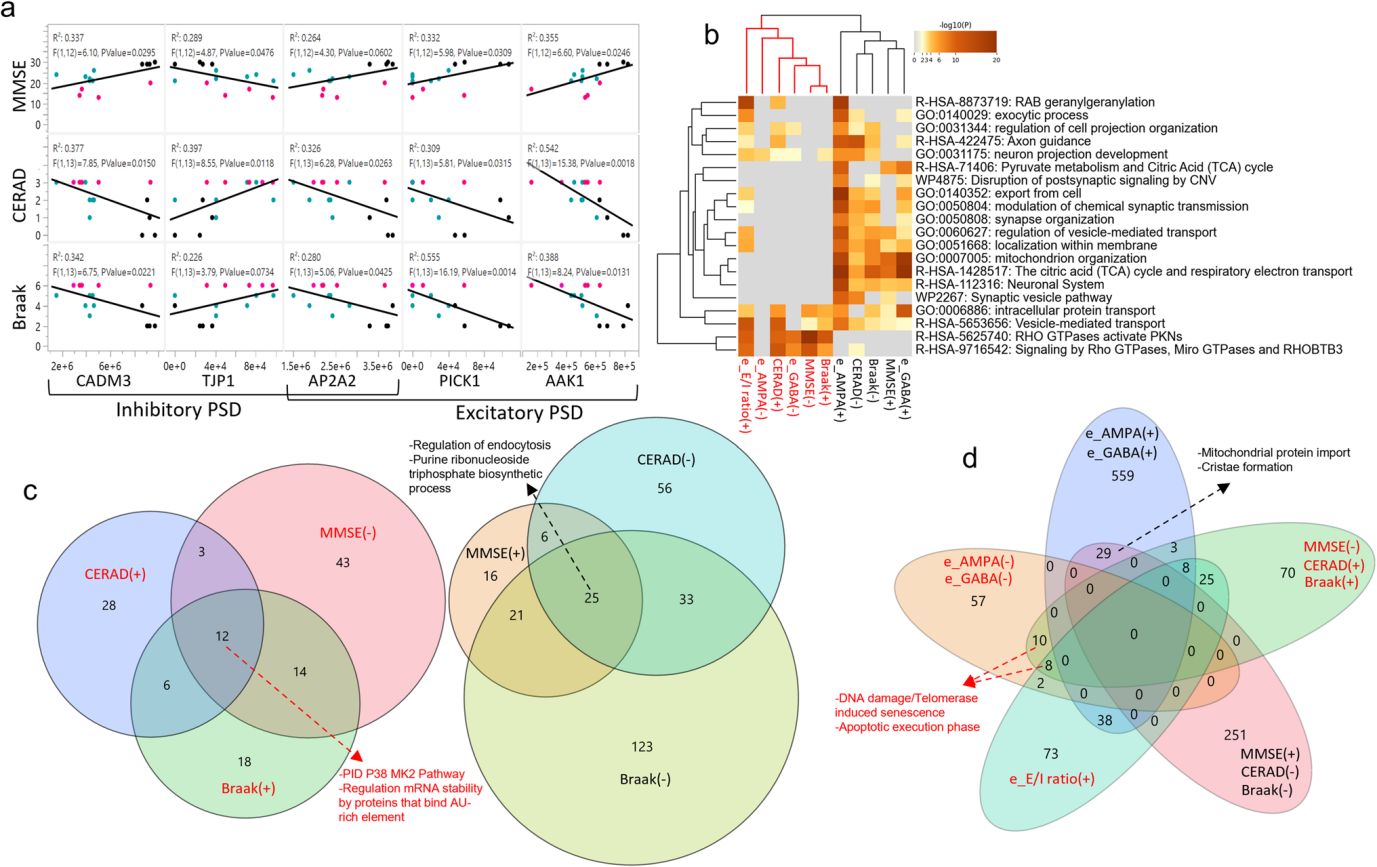
The *e*E/I ratio correlates with neuropathology severity.** a** Linear correlations between inhibitory and excitatory postsynaptic density proteins (PSD) with MMSE scores and severity of AD pathology (Braak stage, CERAD) from CTRL (*n* = 4, black dots), MCI (*n* = 6, aqua dots) and AD (*n* = 6, magenta dots) donors. **b** Heatmap showing the top enrichment clusters, one row per cluster, using a discrete color scale to represent statistical significance. Gray color indicates a lack of significance. Metascape GO enrichment analysis of synaptic proteomics that correlate positively (+) or negatively (−) with MMSE, Braak stage, CERAD, *e*AMPA, *e*GABA, and *e*E/I ratio shows two separated clusters related to the support of synaptic function (text color black) or toxicity (text color red). **c** Venn diagram of synaptic proteins correlating with at least one of the following variables: Braak, CERAD, and MMSE. Proteins correlating with all three variables from the synaptic function cluster are involved in regulation of endocytosis and purine ribonucleoside triphosphate biosynthetic process. Proteins in the toxicity cluster are involved in the P38 MK2 pathway, and regulation mRNA stability by proteins that bind AU-rich element. **d** Venn diagram incorporating proteins correlated with electrophysiological parameters showing a separation between proteins participating in the sustenance or toxicity of synaptic function, as shown in **b**

We then screened for changes specifically in proteins known to be associated with AMPARs or GABA_A_Rs complexes [79, 86]. We found 7 proteins that were reduced in MCI but not in AD, 6 of which were associated with GABA_A_Rs. We also found 8 proteins that were reduced in MCI and AD, 6 of which were auxiliary to AMPARs (Supplementary dataset 7, online resource). Interestingly some of these auxiliary proteins also correlated with MMSE scores and with metrics of neuropathology (Fig. 3a), linking their abundance with synaptic function and cognitive performance. We also observed a subset of 124 proteins that correlated positively with neuropathological severity and negatively with MMSE scores; 14.5% of these proteins negatively correlated with the amplitude of AMPARs or GABA_A_Rs (e.g., TJP1), suggesting a deleterious role for synaptic function (Supplementary dataset 9, online resource). Notably, the larger the amount of APP and total tau measured by proteomics in the synaptosomes, the larger the *e*E/I ratio (Fig. 1k), and the larger the *e*E/I ratio, the more variable and lower the MMSE score (Fig. 1k). To integrate all these information, we implemented an unbiased hierarchical cluster analysis using all levels of quantitative data described thus far. This analysis showed a dendrogram with two major branches (Fig. 3b–d; Supplementary datasets 12 and 13, online resource). The first branch contains proteins enriched in endocytosis regulation, purine ribonucleoside triphosphate biosynthetic process, synaptic and mitochondrial complexes and relates to the *sustenance of synaptic function*, formed by proteins positively correlated with the electrophysiological function of synaptic receptors, lower neuropathology and better cognitive performance. The second branch contains proteins enriched in DNA damage/telomerase induced senescence, inflammation, apoptosis, synaptic transmission, regulation mRNA stability processes and relates to *synaptic toxicity*, formed by proteins negatively correlated with synaptic receptor currents, with higher neuropathology and worse cognitive performance. Taken together, these findings indicate that AD neuropathologic changes may affect synaptic protein complexes very early in the pathology, thus compromising the function of AMPARs and GABA_A_Rs needed for the support of cognitive performance in a continuum, and elevations in the TCx *e*E/I ratio may have a direct relationship with worsening of cognitive performance in the disease. Our data also provides evidence that molecular and functional changes are gradual with a large overlap between control, MCI and AD, which is consistent with accumulated evidence that differentiating the clinical construct MCI from controls at one extreme and AD at the other may be challenging [69].

### Transcriptomic analysis of the synaptic E/I ratio in AD

**Fig. 4:**
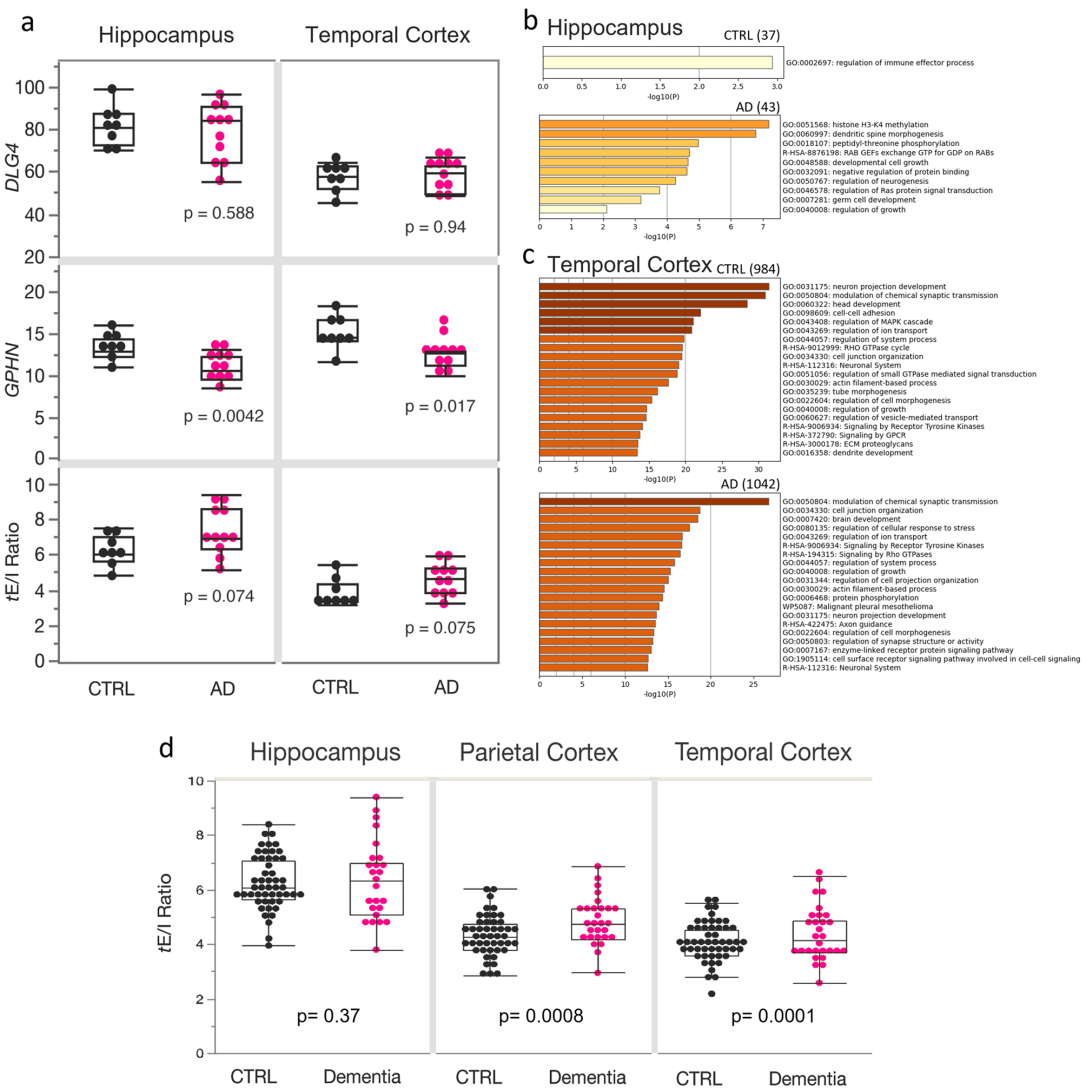
Transcriptomic analysis of excitatory and inhibitory synaptic markers in AD and clinical dementia. **a** RNA-Seq datasets from the ADTBI study show differential alterations in the expression of excitatory (*DLG4*) and inhibitory (*GPHN*) postsynaptic density proteins in the hippocampus and TCx of CTRL and AD (*n* = 8 cognitive healthy CTRL, CERAD = 0 and 12 donors with a DSM-IV clinical diagnosis of dementia of the AD disease type and AD pathology CERAD = 3). The mRNA level of *GPHN* was significantly decreased in AD donors, both in the hippocampus (*F* (1, 18) = 10.95, *p* = 0.0042) and TCx (*F* (1, 18) = 7.06, *p* = 0.0166). No changes were found in *DLG4* expression levels in AD donors in the hippocampus (*F* (1, 18) = 0.006, *p* = 0.9376) and TCx (*F* (1, 18) = 0.31, *p* = 0.5877). In both brain regions, the *t*E/I ratio showed a trend toward pro-excitatory changes in AD (hippocampus: *F* (1, 18) = 3.63, *p* = 0.0739; TCX: *F* (1, 18) = 3.59, *p* = 0.0752). **b**, **c** GO analysis of genes that positively correlate (threshold *p* = 0.001) with the *t*E/I balance. The numbers in parentheses show the number of genes in each group that correlate positively with the tE/I ratio. In the AD hippocampus, the *t*E/I balance was less representative of synapses than the TCx. **d** The ratio between the expression of DLG4 and GPHN in 56 CTRL and 30 AD subjects in the ADTBI cohort. A two-sided *T* test was used to compare levels of expression across brain regions. Boxes in **a** and **d** extend from the 25th to 75th percentiles, and the whiskers extend to 1.5*IQR (points behind the whiskers are considered outliers)

Our proteomic data from synaptic preparations indicated that there were alterations of local protein translation and traffic of proteins in MCI and AD; thus, we evaluated whether an imbalance of the synaptic *e*E/I ratio in the TCx of AD individuals may be observed at the mRNA level. We analyzed the synaptic transcriptional E/I ratio (*t*E/I ratio), defined as the level of mRNA for PSD95 (*DLG4*) to the level of mRNA for gephyrin (*GPHN*) [44], using a publicly available RNAseq dataset from the ADTBI study [105]. For this analysis, we first focused on the hippocampus and TCx of non-demented CTRL donors with no pathology (*n* = 8) and donors with AD neuropathologic change defined as tau neurofibrillary degeneration and neuritic plaque density (*n* = 12; Supplementary dataset 3, online resource), which are well-defined opposite extremes of AD pathology. The expression levels of the inhibitory marker *GPHN* were significantly decreased in both the hippocampus and TCx of donors with AD (Fig. 4a, Supplementary dataset 19, online resource). By contrast, expression levels of *DLG4* in donors with AD were similar to the CTRL donors for both regions (Fig. 4a). The *t*E/I ratios showed a trend toward pro-excitatory changes in AD in both the hippocampus and TCx. To explore which genes had a potential effect on the *t*E/I ratio, we screened the whole gene expression dataset. We found 20-fold more genes correlating with the *t*E/I ratio in the TCx than in the hippocampus (cutoff *p* = 0.001; Supplementary dataset 14, online resource). In TCx, 984 genes in CTRL and 1042 in AD correlated with the *t*E/I ratio (Fig. 4c). GO analyses showed that those genes expressed proteins involved in synaptic function (Supplementary dataset 15, online resource). The hippocampus showed only 37 genes for CTRL and 43 for AD that correlated with the *t*E/I ratio, likely reflecting a highly heterogeneous and variable region across individuals and thus low output on GO analysis (Fig. 4b). Interestingly, a larger number of genes were correlated with *t*E/I in the temporal cortex of AD donors than in controls, and the “modulation of chemical synapses” was the most enriched GO term in AD. This transcriptomic profile was congruent with the differences observed at the proteomic levels between AD and control synaptosomes, where a larger number of proteins correlated with AMPARs and GABA_A_Rs responses in AD than in control, and a stronger enrichment of synaptic processes were observed in AD.

It is important to note that the donors with AD in this first analysis had abundant neuropathology, whereas CTRL donors had minimal, if any (CERAD = 0), which excludes a large diversity in the continuum of symptomatic AD cases in the population. Thus, we repeated the analysis using transcriptomic data on all available 56 CTRL and 30 AD donors of the ADTBI cohort (Supplementary dataset 3, online resource), excluding only demented individuals due to vascular, multiple etiology, or unknown causes. This larger cohort also includes resilient people with high AD neuropathology but normal cognitive scores [9, 43], people with MCI and people diagnosed with clinical dementia of the AD type but without sufficient corresponding AD neuropathology [35]. Therefore, this analysis tests whether the *t*E/I ratio is different in people with dementia compared to those without it across a continuum of cognitive performance. We also included PCx data in this analysis [44], which we previously showed has a significantly increased *t*E/I ratio in people with severe AD neuropathologic change and dementia vs CTRL with minimal pathology. We found that RNA integrity, sex and age had no association with the *t*E/I ratio in the hippocampus and TCx, and only the RNA integrity number (RIN) had a small effect on the PCx (Supplementary dataset 16, online resource). Importantly, the *t*E/I ratio was not different in the hippocampus but was significantly increased in the PCx (with [*p* = 0.009] and without correcting for RIN [*p* = 0.008]) and TCx of people with AD dementia (Fig. 4d, Supplementary dataset 19, online resource), indicating that the *t*E/I ratio imbalance in the PCx and TCx may be involved in the development of dementia.

### Pro-excitatory shift of the cellular E/I ratio in AD

**Fig. 5:**
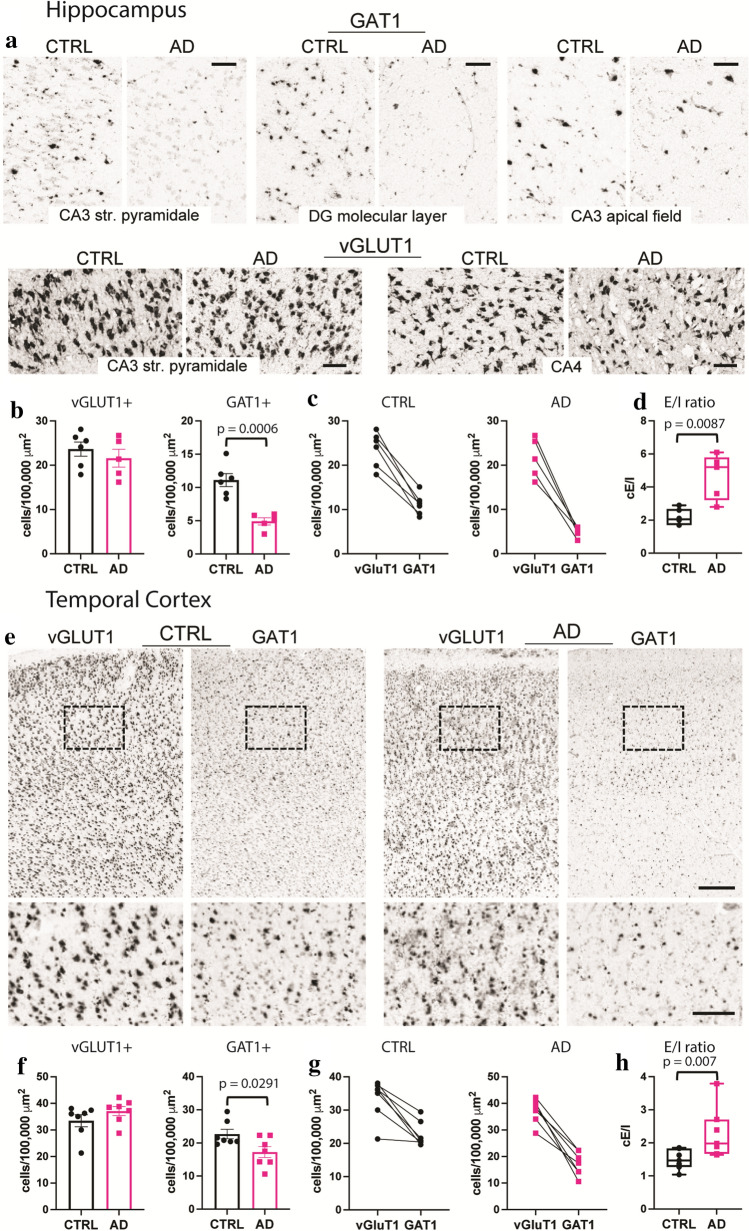
Marked reductions in GAT1 mRNA-expressing cells in AD cases with dementia. **a** Images showing ISH for vGluT1 and GAT1 mRNAs in several hippocampal subfields targeted for analyses from CTRL no dementia-CERAD 0 and dementia-CERAD 3 AD cases. Calibration bars, 100 µm. **b** Quantification of vGluT1 and GAT1 mRNA-expressing cells per 100,000 µm^2^. For vGluT1, measures from the CA3 *stratum pyramidale* and CA4 were averaged, and for GAT1, measures from the CA3 *stratum pyramidale*, CA3 apical dendritic field, and the dentate gyrus molecular layer were averaged (see Methods); counts for individual areas are presented in Supplementary Fig. 10 (online resource). GAT1-expressing cells were reduced in the AD group (*n* = 5) versus the CTRL group (*n* = 6) (*p* = 0.0006, two-tailed unpaired Student’s *t* test). **c** Plots show the relative density of vGluT1 and GAT1 mRNA-expressing cells for each CTRL and AD case and the E/I ratio of the two measures for each case (**d**). The cellular E/I ratio in the hippocampus was significantly elevated in the AD group versus CTRL (**p = 0.0087, two-tailed unpaired Mann–Whitney test). **e** Images showing ISH for vGluT1 and GAT1 mRNAs in TCx of a CTRL no dementia-CERAD 0 case and a dementia-CERAD 3 AD case. The box shows the region that is presented at higher magnification (below). Calibration bars, 500 µm for top images; 200 µm for bottom images. **f** Quantification of vGluT1 and GAT1 mRNA-expressing cells in TCx per 100,000 µm^2^. GAT1-expressing cells were reduced in the AD group (*n* = 7) versus the CTRL group (*n* = 7) (*p = 0.2188, two-tailed unpaired Student’s *t* test). **g** Plots show the relative density of vGluT1 and GAT1 mRNA-expressing cells for each CTRL and AD donor and **h** the E/I ratio of the two measures for each case. The cellular E/I ratio in TCx was significantly elevated in the AD group versus CTRLs (**p = 0.007, two-tailed unpaired Mann–Whitney test). The data shown are based on a 30 µm^2^ size threshold; separate analyses using 10 and 20 µm^2^ size thresholds yielded similar results (Supplementary Fig. 10, online resource). Box plots indicate median values, 25th and 75th percentiles, and minimum and maximum range. Whiskers in bar plots represent standard errors

To determine whether *e*E/I imbalance in the TCx or the lack thereof in the hippocampus was due to alterations in E/I cellular ratios, we used the publicly available in situ hybridization (ISH) image dataset from the ADTBI study [105] and evaluated 20 donors from the same RNA-Seq datasets of the ADTBI cohort at the extremes of AD pathology (8 CTRL donors with CERAD = 0 and 12 AD donors with CERAD = 3). The numbers of glutamatergic neurons that expressed mRNA for the excitatory vesicular glutamate transporter 1 (vGluT1; vGluT1 + _mRNA_) and GABAergic neurons that expressed mRNA for GABA transporter 1 (GAT1; GAT1 + _mRNA_) were quantified for several subfields of the hippocampus and TCx layers I–VI (Fig. 5, Supplementary dataset 19, online resource). For these analyses, vGluT1 + _mRNA_ and GAT1 + _mRNA_ cells were counted in defined sample fields, and their densities (number of cells/area) were compared between the AD and CTRL groups. For cellular E/I (*c*E/I) measurements within the hippocampus, several laminae were analyzed for either vGluT1 + _mRNA_ or GAT1 + _mRNA_ cells, and regional analyses were combined. Thus, excitatory vGluT1 + _mRNA_ cells were counted in sample fields of the CA3 *stratum pyramidale* and the dentate hilar region referred to as CA4, and inhibitory GAT1 + _mRNA_ cells were counted in the CA3 *stratum pyramidale*, CA3 apical dendritic field, and the dentate gyrus molecular layer (Fig. 5a, Supplementary Figs. 9, 10, online resource). For each mRNA, the counts across all sample fields were averaged per case; this sampling strategy was used to assess changes in the two cell types because inhibitory cells are more widely distributed across hippocampal subfields, while excitatory cells are concentrated in specific laminae. Densities of vGluT1 + _mRNA_ cells were not different between groups, whereas the densities of GAT1 + _mRNA_ cells were significantly reduced in AD (Fig. 5b, c). Comparing the numbers of hippocampal vGluT1- to GAT1-expressing cells for each case demonstrated a marked increase in the *c*E/I ratio in the AD group (Fig. 5d). In TCx, where both mRNAs were assessed within the same matched sample field (Fig. 5e), densities of vGluT1 + _mRNA_ cells were not different between groups, whereas densities of GAT1 + _mRNA_ cells were significantly reduced in AD (Fig. 5f, g). Thus, as in the hippocampus, the TCx exhibited a marked increase in this *c*E/I ratio in the AD group (Fig. 5h). Taken together with the RNA-seq data, the findings support the conclusion that there are marked widespread decreases in the expression of GABAergic markers that contribute to the E/I imbalance in AD.

### Multilevel pro-excitatory changes in the E/I ratio in AD correlate with loss of cognitive performance

**Fig. 6:**
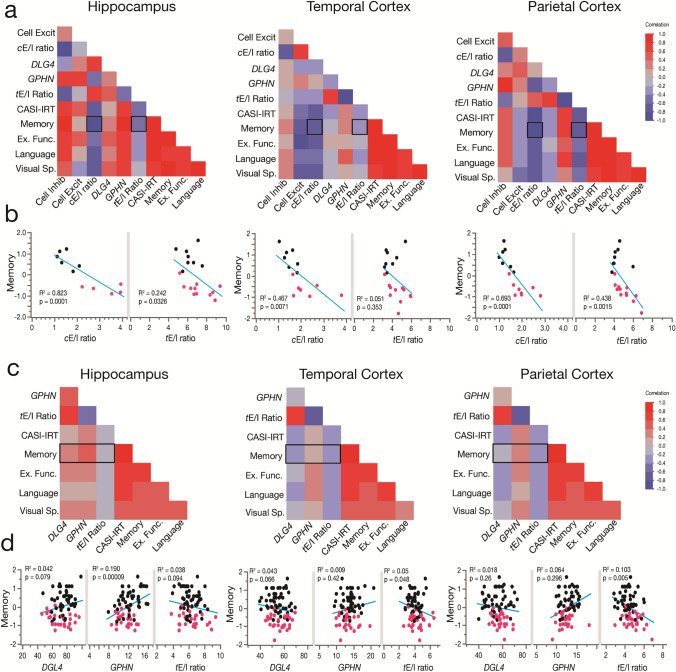
The cellular and synaptic E/I balance in the hippocampus, TCx, and PCx correlates with cognitive performance. **a**, **b** Weighted multivariate analysis showing correlation maps for cognitive assessments of CTRL (*n* = 8) and AD (*n* = 12) individuals with cellular and synaptic excitatory and inhibitory markers and their ratio from the hippocampus, TCx and PCx. Individuals were scored with the Cognitive Abilities Screening Instrument calibrated using item response theory (CASI-IRT), memory, executive function (Ex. Func), language and visuospatial performance (Visual Sp.). See the cognitive scores section for more information about the use of weight in the analyses. The hippocampus, TCx and PCx showed cellular (*c*E/I) and transcriptional synaptic E/I (*t*E/I) ratios correlated with the memory performance of the individuals (*p* values from Pearson’s correlations). **c**, **d** Pearson’s correlation matrix showing cognitive assessments of 86 individuals (56 nondemented, 30 demented AD type) and RNA-seq data of synaptic excitatory (*DLG4*) and inhibitory (*GPHN*) markers and their ratio. Hippocampus (*n* = 75 donors), TCx (*n* = 79 donors) and PCx (*n* = 74 donors) (not all the donors had data from the three brain regions). In the hippocampus, memory showed a significant correlation with a reduction in inhibitory synaptic markers and a trend with a loss of excitatory synaptic markers. Cortical regions showed that memory loss better correlates with *t*E/I ratio increases

Our electrophysiological results suggest that larger cortical *e*E/I ratios are correlated with worse MMSE scores and higher neuropathology. Therefore, we first evaluated 20 donors from the same RNA-Seq datasets of the ADTBI cohort at the extremes of AD pathology (8 CTRL donors with CERAD = 0 and 12 AD donors with CERAD = 3) to test whether larger *c*E/I and *t*E/I ratios correlate with cognitive impairment. Cognitive scores from the Cognitive Abilities Screening Instrument using Item Response Theory (CASI-IRT) [17], as well as the most recent previously calibrated composite scores for memory, executive function, language, and visuospatial abilities were obtained as described before [18]. Item Response Theory (IRT) addresses the limited sensitivity at higher levels of cognitive functioning and nonlinear measurement properties of the CASI, and unlike standard total scores, IRT scores have linear scaling properties providing a better representation of the different levels of cognitive performance in the cohort [45]. We also included PCx data in this analysis [44]. Our multivariate weighted analysis showed strong negative correlations between the degree of pro-excitatory shift of the *c*E/I in the three brain regions and cognitive scores (Fig. 6a, b). For example, *c*E/I in the hippocampus, TCx and PCx explained 82, 47 and 69% of the variance in memory scores, whereas *t*E/I explained 24, 5 and 44% of the variance, respectively (Supplementary dataset 17, online resource). These results indicate that dementia correlates with cellular and transcriptional E/I imbalance differently across brain regions. We repeated the analysis using transcriptomic data on all 56 available CTRL and 30 AD donors of the ADTBI cohort (Supplementary dataset 3, online resource), excluding only demented individuals due to vascular, multiple etiology, or unknown causes. We did not use the *c*E/I ratio because these data were not available for all donors. We instead used the *t*E/I, which explains less of the variance compared to *c*E/I but which was available for the entire cohort. Importantly, the *t*E/I ratio in the PCx and TCx of people with AD dementia (Fig. 4d) was negatively correlated with cognitive scores (Fig. 6c, d). In contrast, in the hippocampus, the *t*E/I ratio was not different across diagnoses and was not correlated with cognitive scores. However, on average, lower levels of both inhibitory and excitatory markers were associated with lower cognitive scores (Fig. 6c, d). These results mirror our findings using electrophysiological data and MMSE scores. To further evaluate the role of regional levels of pathology and the *t*E/I ratio on cognitive abilities, we used Luminex protein data from the same region/donors in stepwise linear regression analyses (Supplementary dataset 18, online resource). Table 1 shows the two most significant factors explaining cognitive scores and the variance explained across brain regions, which increased by inclusion of pathology markers. For TCx and PCx, the *t*E/I was the strongest factor for cognitive (CASI-IRT scores), memory and executive function.

**Table 1:** Multiple linear regression of top factors on cognitive performance

Brain region	Outcome	Effect	LogWorth	*p* value	*R*^2^
Hippocampus	CASI-IRT	Ihc at8	2.59	0.0026	0.30
		Ihc Aβ ffpe	2.50	0.0031	
	mem	Aβ_42_/Aβ_40_ ratio	2.52	0.0031	0.22
		ihc at8	0.85	0.1420	
	exf	ihc tau2 ffpe	4.15	< 0.0001	0.32
		Il-1β pg/mg	2.04	0.0091	
	lan	ihc at8 ffpe	3.91	0.0001	0.55
		ihc iba1 ffpe	2.82	0.0015	
	vsp	mcp1 pg/mg	2.99	0.0010	0.62
		ihc gfap ffpe	2.82	0.0015	
TCx	**CASI-IRT**	***t*E/I ratio**	**2.28**	**0.0052**	0.15
		Aβ_1-42_ pg/mg	1.36	0.0438	
	mem	ihc Aβ	1.17	0.0675	0.09
		tE/I ratio	0.79	0.1618	
	**exf**	***t*E/I ratio**	**1.96**	**0.0111**	0.10
		mip1a pg/mg	0.55	0.2797	
	lan	ihc at8 ffpe	4.57	< 0.0001	0.39
		Ihc gfap ffpe	2.56	0.0027	
	vsp	ihc at8	1.61	0.0248	0.21
		Aβ_1–40_ pg/mg	1.56	0.0275	
PCx	**CASI-IRT**	***t*E/I ratio**	**4.21**	**< 0.0001**	0.46
		Isoprostane pg/mg	2.92	0.0012	
	**mem**	***t*E/I ratio**	**3.65**	**0.00022**	0.43
		ihc Aβ	2.90	0.0013	
	**exf**	ihc Aβ ffpe	5.87	< 0.00001	0.70
		***t*E/I ratio**	**5.77**	**< 0.00001**	
	lan	ihc Aβ	5.39	< 0.00001	0.51
		ihc α-syn	4.00	0.0001	
	vsp	Aβ42 pg/mg	3.62	0.0002	0.39
		ihc Aβ	3.34	0.0005	

Outcome, Y variable; CASI-IRT, cognitive scores from the Cognitive Abilities Screening Instrument using Item Response Theory; mem, exf, lan, vsp, normalized metrics for memory, executive function, language, and visual spatial performance, respectively; Effect, *X* variable; LogWorth, − log10(*p* value). *R*^*2*^, variation explained by the model; TCx, temporal cortex; PCx parietal cortex. Ihc *Z*, % of area covered by *Z* immunoreactivity on fresh tissue; Ihc *Z* ffpe, % of area covered by *Z* immunoreactivity on formalin-fixed, parafilm-embedded tissue. AT8, monoclonal antibody recognizing tau protein phosphorylated at ser202 and thr205; Tau2 reacts with 52–68 kDa tau in phosphorylated and non-phosphorylated forms; Il 1b pg/mg, interleukin 1b; ibai, ionized calcium-binding adaptor molecule; mcp1 pg/mg, monocyte chemoattractant protein-1; gfap, glial fibrillary acidic protein; Aβ_1–42_ pg/mg, 42 amino-acid beta-amyloid peptide derived from the amyloid precursor protein (*APP*); mip1a pg/mg, macrophage inflammatory protein-1 alpha; Aβ_1–40_ pg/mg, 40-amino acid beta-amyloid peptide derived from APP; Isoprostane pg/mg, prostaglandin-like compounds produced by the reaction of free radicals with arachidonic acid; α-syn, α-synuclein. Bold letters highlight the cases where *t*E/I ratio effects were significant. See data for all effects in Supplementary dataset 18 (online resource)

## Discussion

Growing evidence implicates hyperexcitability in AD with synaptic E/I imbalance [51]. High synaptic activity induces perisynaptic release of tau and Aβ proteins [16, 70, 95], and although the role of tau oligomers in neuronal excitability is still a matter of continuous investigation [37], soluble oligomeric forms of Aβ increase synaptic activity [12, 102], suggesting that a positive feedback loop between hyperexcitability and amyloidosis may first compromise neuronal function and then lead to neurodegeneration. However, whether pro-excitatory synaptic changes are observed in brain areas affected early in AD and whether they are emergent in MCI is not known. Another important unresolved question is whether global synaptic E/I imbalance correlates with the severity of cognitive impairment in the continuum of AD clinical syndrome. Here we address these questions, in two brain regions critical for the continuum of AD neuropathological change, by analyzing the correlations between synaptic markers across different levels of analysis and MMSE and CASI-IRT scores.

### Cortical synaptic dysfunction and imbalance in MCI and AD

Non-injected Xenopus oocytes do not produce AMPA or GABA_A_Rs currents [44, 97], therefore agonist-induced responses in oocytes injected with synaptic membranes result only from the activation of microtransplanted synaptic receptors that were isolated from the brains of non-demented, MCI or AD brains. These receptors include potential alterations found in the synaptic microenvironment associated with each diagnostic condition [75]. We found that the resting membrane potential was more depolarized in oocytes microtransplanted with synaptosome-enriched P2 fractions from AD compared to MCI but not with control donors. P2 fractions, although mainly enriched with synaptosomes, may also contain free mitochondria, vesicles, myelin, and other membrane fragments that could significantly differ between groups. In AD, soluble Aβ oligomers (oAβ) have been detected in P2 fractions, especially at early stages of the disease [8], while in MCI, the mean value of oAβ lies between that obtained from AD and control individuals [26]. Such oAβs form ion channel pores that disrupt intracellular Ca^2+^ homeostasis [10, 78] and thus may explain why the resting membrane potential in oocytes with AD membranes was more depolarized than that in oocytes with MCI membranes. The large variability in the resting potential of oocytes microtransplanted with membranes from control individuals, possibly due to interindividual differences [48] and distinct effects of agonal factors [84], may have obscured differences between the resting potential of oocytes microtransplanted with control and AD membranes.

Our electrophysiological analysis found that the relationship between the responses of AMPARs and GABA_A_Rs in AD are distinct in the hippocampus compared to the TCx. While we did not find an imbalance of the *e*E/I ratio in hippocampus of AD donors, we did find a marked increase of the *e*E/I ratio in the TCx of AD individuals that was significantly different from control but not from MCI donors, suggesting that MCI presents changes that are intermediate between control and AD. These gradual changes in MCI were confirmed by an early increase of proteins that promote tau phosphorylation, and a reduction of synaptic and mitochondrial complexes in MCI. The early increase of proteins involved in tau phosphorylation is in agreement with recent findings showing that AD symptom onset is associated with stronger tau pathology [24] and suggests that early tau pathology may be driving some of the synaptic changes observed in MCI. The increase of the *e*E/I ratio in AD was not driven by changes in the affinity of AMPARs or GABA_A_Rs but by (i) a gain of function of AMPARs as seen by the higher slope between AMPA vs GABA responses in AD and which may be related to posttranslational modifications due to the effects of oAβ [23], and (ii) alterations in the organization of GABAergic synaptic complexes. Whereas the amplitude of AMPA currents largely correlated with excitatory synaptic and postsynaptic density proteins, the amplitude of GABA currents did not correlate with inhibitory synaptic complexes or other inhibitory markers. This is in line with the dissociation between synaptic GABA_A_Rs and inhibitory postsynaptic density proteins observed in AD [1, 30, 47].

The *e*E/I ratio in the hippocampus, although largely variable, was surprisingly not different among groups, indicating that synaptic GABA responses were proportional to those of AMPA. Additionally, we did not find differences in the total number of labeled PSD95 or gephyrin-immunolabeled synaptosomes in the hippocampus, even though marked reductions in the total number of synapses have been reported for AD and MCI in CA1 using transmission electron microscopy [61] and in the CA1 and CA3 *stratum pyramidale* by immunofluorescence [25]. This suggests that our approach may not have sufficient resolution to detect subfield changes in the hippocampus or that the affected areas may be potentially compensated for neurogenesis [36] or other neuroplastic changes [57]; other possibilities also include large hippocampal heterogeneity [15, 27] and interindividual variability [27, 71, 79, 87]; indeed, twin studies have shown that the hippocampus is the least heritable brain region [67], indicating a high capacity for remodeling and plasticity compared to cortical regions. Interestingly, the number of labeled PSD95 synaptosomes in hippocampus, and as mentioned before, the resting membrane potential of oocytes microtransplanted with hippocampal and cortical synaptosomes showed group differences between AD and MCI, but not controls with MCI, or controls with AD. Because MCI has lower values for these parameters than controls and AD, the expression pattern resembles an inverted pyramid, which is an MCI pattern that has also been observed in regional gene expression in a microarray analysis [6]. Therefore, we cannot exclude the possibility that along the AD pathology continuum both hippocampus and cortical regions may go through several processes of pathology and synaptic compensatory mechanisms, where a subset of genes and proteins in MCI may have a unique signature, but others show a gradual overlap with controls and AD. Our synaptic results in the hippocampus are in line with previous 3D electron microscopy studies demonstrating preservation of the synaptic E/I ratio in CA1 of AD donors [55]. We were unable to directly assess CA1, a hippocampal region affected early on in AD [11], but found marked interneuron loss in CA3 and CA4 in our ISH analysis. Thus, despite inhibitory neuronal loss in the disease, the relative abundance between excitatory and inhibitory synapses is likely maintained by a compensatory increase in the expression of GABA_A_ receptors as recently reported [42].

### Correlation between cortical synaptic imbalance and cognitive performance

We previously presented evidence for a shift of the global E/I synaptic balance in the PCx of individuals with early-onset AD [44]; however, to the extent of our knowledge, whether global synaptic E/I imbalances correlate with the severity of cognitive impairment has not been previously evaluated.

Our data showed that the *e*E/I ratio in the hippocampus was not different in MCI or AD; however, lower amplitudes of GABA_A_Rs and AMPARs currents in the hippocampus were associated with lower cognitive scores, indicating that hippocampal atrophy, with total loss of excitatory and inhibitory synapses, correlates better with cognitive impairment, as observed in hippocampal sclerosis studies [80]. This is also in agreement with strong evidence supporting the relationship between positive modulation of hippocampal AMPARs [50, 56, 68, 93] with better cognition. In contrast, we found that a gain of function of AMPARs in parallel with GABAergic deficits lead to a striking pro-excitatory shift of the E/I balance in the TCx of AD individuals across various levels of analyses. Consistent with the progressive nature of AD, the pro-excitatory *e*E/I ratio in the TCx was positively correlated with higher levels of AD biomarkers (tau, Braak stage, APP, and CERAD score) and with cognitive impairment. Our proteomic analysis in the TCx found a large number of synaptic proteins critical for neuronal functioning that correlated with cognitive performance and AD neuropathology. Some of them were already known to play a role in AD: PICK1 is involved in synaptic scaling [3]; AP2A2 is involved in receptor-mediated endocytosis [60]; SYT7 is regulated by amyloid precursor protein [5]; and KIF5B is critical for GABA_A_R transport, and its deletion causes epilepsy [58]. Together, our proteomic findings using the function of receptors as an anchor for its analysis support the role of synaptic function in cognition and its gradual loss with progressive cognitive impairment and dementia. Furthermore, we were able to correlate E/I imbalance at different levels (*e*E/I, *p*E/I, *t*E/I and *c*E/I ratios) with the cognitive status of the donors in this study. The robust pro-excitatory shift of E/I balance associated with loss of cognition and pathology severity suggests that there is a strong connection between these events. Thus, stability of the E/I ratio is a critical property of the cerebral cortex, and loss of this balance could potentially lead to neuronal and circuit dysfunction leading to a clear hyperexcitability first and a progressive slowing of the EEG power and hypometabolism as AD neuropathology progresses. Interestingly, our results indicate that MCI presents gradual synaptic modifications between controls and AD; however, functional imaging shows a strong hyperactivity in MCI. One possibility is that hyperactivity in early MCI results primarily from the effects of soluble amyloid oligomers that enhance excitatory inputs in primary cells by the direct activation of AMPA and NMDA receptors [2, 83], and the blockade of glutamate synaptic recapture that enhances preexisting excitatory activity [102]. This hyperexcitability may then be followed up by maladaptive homeostatic mechanisms that leads to the gradual functional and structural synaptic changes across the continuum of AD. It is important to note that since our study is not longitudinal, there are time-dependent components in circuit excitability that we cannot address. However, slowing of the EEG in AD does not necessarily mean general circuit hypoexcitability. While the power of high frequency waves is reduced, there is an increase of theta power [19] which is known to strongly promote synaptic potentiation [85], our data suggests that despite synapse loss in MCI and AD, the remaining synapses have larger currents, suggesting increased excitatory synaptic activity which cannot be compensated by faulty inhibitory activity. Indeed, hypometabolism, measured as reduction of FDG-PET, is a common finding with strong clinical utility in patients with temporal lobe epilepsy [33], suggesting that hypometabolism arises from neuronal damage but also may be due to energetic alterations due to an initial high excitability demand.

The role of hyperexcitability in cognitive impairment is highlighted by accumulated evidence showing that positive allosteric modulators [32, 39, 72, 74] of GABA_A_Rs have been associated with pro-cognitive activity [72] and with reduced Aβ production and decreased pathological features of AD [46, 98]. Positive drug effects on AMPAR and GABA_A_Rs agree with our findings in the TCx: the observed shift in the E/I balance is mostly due to a decrease in inhibitory neurons, and low GABA currents are associated with worse cognitive scores, suggesting that GABAergic stimuli should be associated with better cognition. Recently, a retrospective study observed a lower prevalence of AD in individuals prescribed with bumetanide for blood pressure control compared to those that were prescribed a different drug, suggesting that bumetanide, which has strong effects on GABAergic signaling, may be beneficial; however, double-blind placebo control studies are still needed to determine the clinical importance of these results [82, 97]. Bumetanide was also shown to rescue electrophysiological, pathological, and cognitive deficits in an AD mouse model through a pathway linked to GABAergic synapses [82]. Similarly, antiepileptic drugs have been shown to be beneficial to people with AD, including having positive effects on cognitive function in the disease [20, 66, 90]. Given that the global synaptic E/I ratio positively correlates with neuronal firing [31], it may also be the case that treatments that control aberrant hyperactivation in specific brain regions in AD could also be of value during the prodromal stages of the disease. Taken together, our results support the general idea that correcting the E/I imbalance through a GABAergic mechanism may be an effective, disease-modifying approach for the prevention and treatment of AD.

## Supplementary Information

Below is the link to the electronic supplementary material.

Supplementary file1 (XLSX 6928 kb)

Supplementary file2 (XLSX 13 kb)

Supplementary file3 (XLSX 286 kb)

Supplementary file4 (XLSX 43 kb)

Supplementary file5 (XLSX 14 kb)

Supplementary file6 (XLSX 11 kb)

Supplementary file7 (XLSX 353 kb)

Supplementary file8 (XLSX 50 kb)

Supplementary file9 (XLSX 15 kb)

Supplementary file10 (XLSX 36 kb)

Supplementary file11 (XLSX 104 kb)

Supplementary file12 (XLSX 147 kb)

Supplementary file13 (XLSX 211 kb)

Supplementary file14 (XLSX 67 kb)

Supplementary file15 (XLSX 881 kb)

Supplementary file16 (XLSX 295 kb)

Supplementary file17 (XLSX 9 kb)

Supplementary file18 (XLSX 24 kb)

Supplementary file19 (XLSX 14 kb)

Supplementary file20 (PDF 2252 kb)
