# Supplementary material for: Functional excitatory to inhibitory synaptic imbalance and loss of cognitive performance in people with Alzheimer’s disease neuropathologic change
Source: Acta Neuropathol. Author manuscript; Available in PMC 2023 Mar 8. (PMC9925531; doi:10.1007/s00401-022-02526-0)
Supplement: 1870757_Supplementary FIgures [file NIHMS1870575-supplement-1870575_Sup_file_20.pdf]

Excitatory to inhibitory imbalance and loss of cognitive performance in people with Alzheimer's disease neuropathologic change

Pietro Scaduto, Julie C. Lauterborn, Conor D. Cox, Anna Fracassi, Tommaso Zeppillo, Berenice A. Gutierrez, C. Dirk Keene, Paul K. Crane, Shubhabrata Mukherjee, William K. Russell, Giulio Taglialatela, Agenor Limon.

Supplementary Figures

Supplementary Figure 1. Synaptosome fraction quality control

Supplementary Figure 2. Flow cytometry size-gated synaptosome in hippocampus.

Supplementary Figure 3. Flow cytometry size-gated synaptosome in temporal cortex.

Supplementary Figure 4. Correlations between electrophysiological parameters and MMSE scores.

Supplementary Figure 5. Correlation between synaptic excitatory and inhibitory currents.

Supplementary Figure 6. Metascape analysis of proteins differentially expressed across groups.

Supplementary Figure 7. Proteomic analysis of excitatory and inhibitory synaptic receptors and markers.

Supplementary Figure 8. Metascape analysis of proteins positively correlated with electrophysiological responses of AMPARs and $GABA_A$Rs.

Supplementary Figure 9. Representative low power in situ hybridization for vGluT1 and GAT1 mRNAs

Supplementary Figure 10. In Situ hybridization analysis using different size thresholds.

Supplementary datasets

Supplementary Dataset 1. Demographics University of California Irvine Cohort.

Supplementary Dataset 2. Proteomics synaptic enrichment using SynGO

Supplementary Dataset 3. Demographic and clinical data of ADTBI cohort.

Supplementary Dataset 4. Electrophysiological responses data.

Supplementary Dataset 5. Concentration response data.

Supplementary Dataset 6. Proteomic analyses dataset.

Supplementary Dataset 7. Differential proteomic expression in TCx by diagnosis

Supplementary Dataset 8. Metascape top enriched clusters of differentially expressed proteins across MCI, AD and controls.

Supplementary Dataset 9. List of proteins positively and negatively correlated with MMSE, Tangle, CERAD, and electrophysiological responses from AMPARs or $GABA_A$Rs or both.

Supplementary Dataset 10. Metascape of proteins positively correlated with electrophysiological responses of AMPAR and $GABA_A$R (Supplementary Figure 8a).

Supplementary Dataset 11. Metascape of proteins positively correlated with electrophysiological responses of AMPAR and $GABA_A$R (Supplementary Figure 8b, c).

Supplementary Dataset 12. Metascape top enrichment clusters heatmap of figure 3b of proteins correlating differentially with MMSE, Tangles and CERAD in the TCx, including electrophysiological data.

Supplementary Dataset 13. Metascape top enrichment bar graph of figure 3c-d of proteins correlating differentially with MMSE, Tangles and CERAD in the TCx, including electrophysiological data.

Supplementary Dataset 14. Expressed genes correlated with *t*E/I ratio.

Supplementary Dataset 15. Metascape of expressed genes correlated with the *t*E/I ratio.

Supplementary Dataset 16. effects of RIN, sex and age on *t*E/I ratio.

Supplementary Dataset 17. Multivariate correlation analysis data.

Supplementary Dataset 18. Linear regression minimum Bayesian Information Criterion (BIC) equation data.

Supplementary Dataset 19. Mean values of Fig. 4, 5, Supplementary Fig. 7, 10.

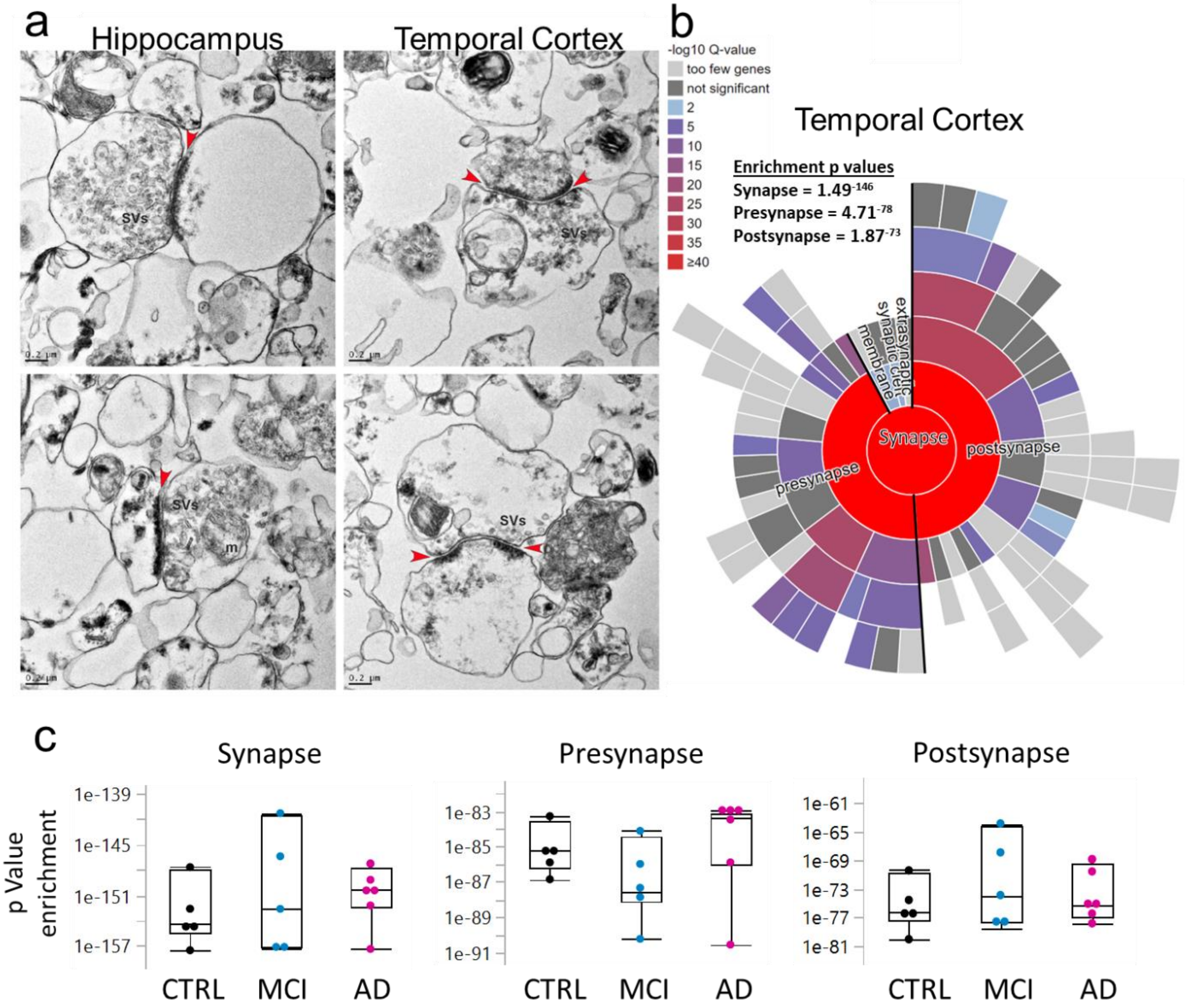

Supplementary Figure 1. Synaptosome fraction quality control. **a,** Representative electron micrographs of synaptosomes fraction (P2 fraction) from human hippocampus (left) and temporal cortex (right). The images show presynaptic terminals with synaptic vesicles (SVs) and, postsynaptic compartments with electron dense postsynaptic regions (red arrowheads). m, mitochondria. Scale bar 0.2 µm. **b,** P2 fraction proteomics was analyzed using SynGO database (https://www.syngoportal.org) and background geneset "brain". The analysis showed high enrichment of pre- and post-synaptic terms. 716 / 2902 proteins from P2 fraction were mapped to unique SynGO annotated genes (detailed info in Supplementary Dataset 2). **c,** Enrichment p values for synapse, presynapse, and postsynapse did not show differences within diagnostic groups (one-way ANOVA, synapse $F\ (2, 15) = 1.12$, $p = 0.357$; presynapse $F\ (2, 15) = 2.72$, $p = 0.103$; postsynapse $F\ (2, 15) = 1.12$, $p = 0.357$). Each dot represents a single subject.

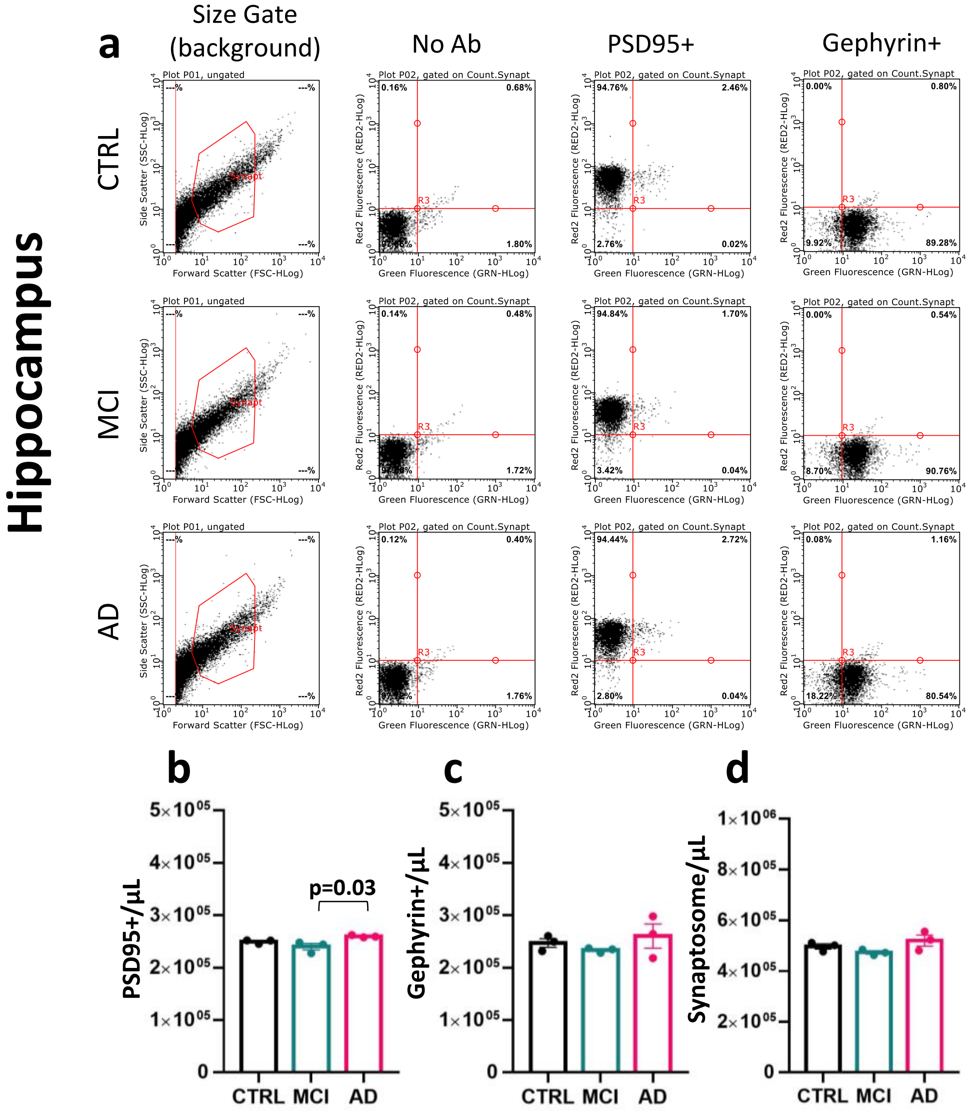

Supplementary Figure 2. Flow cytometry size-gated synaptosome counting in hippocampus. a, Representative flow cytometer plots of pooled synaptosomes from the hippocampus of 8 CTRL, 8 MCI and 11 AD subjects (each point is a technical replicated of pooled synaptosomes per each group). Synaptosome particles were labeled with anti-PSD95 (1:80, Novus-NB300-556AF647) or anti-Gephyrin (1:100, Abcam-Ab32206) and size gated using side scatter/forward scatter. Size gates were built using standardized size beads (Spherotech Inc., gate size from 1 to 3 µm) to include synaptosome size-like particles and excluding background particles. **b, c,** Number of synaptosome-like particles positive for PSD-95 ($PSD95^+$) were not different between MCI or AD compared to CTRL (one-way ANOVA, F (2, 6) = 6.28, p = 0.03 followed by Dunnett multiple comparison test MCI, p = 0.26; AD, p = 0.25), however, MCI was reduced compared to AD (Tukey multiple comparison AD vs MCI, p = 0.03). **c,** Number of size-gated synaptosomes positive for gephyrin ($gephyrin^+$) did not change across diagnosis groups (one-way ANOVA, F (2, 6) = 0.89, p = 0.46). **d,** Total number of synaptosomes positive to PSD95 and gephyrin did not show significative change across diagnostic groups (one-way ANOVA F (2, 6) = 2.57, p = 0.16). Mean values of particles positive for $PSD95^+$ per microliter for CTRL, 249495; MCI, 239686; AD, 259436. For $gephyrin^+$, CTRL, 246892; MCI, 233331; AD, 260018. Total number of particles is the sum of $PSD95^+$ plus $gephyrin^+$: CTRL, 496387; MCI, 473017; AD 519454. Whiskers represent standard error.

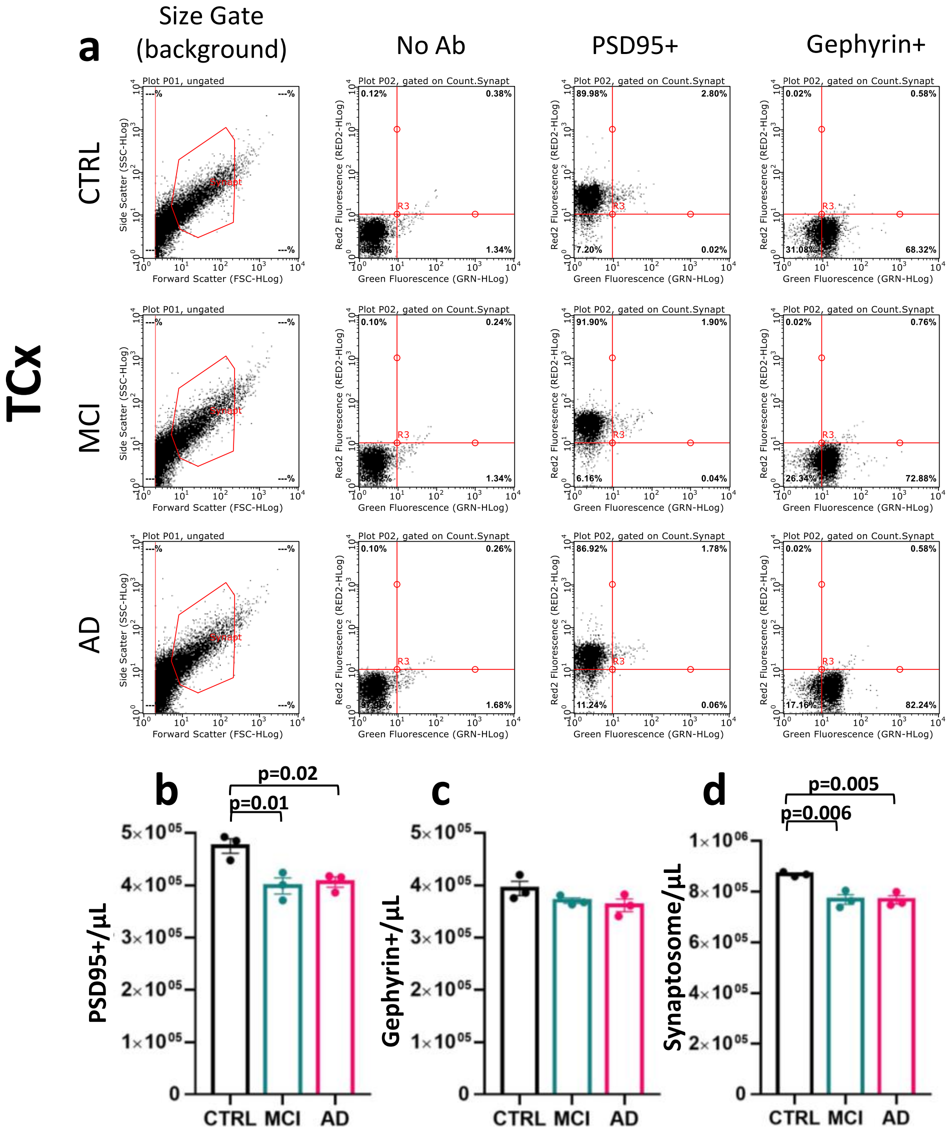

Supplementary Figure 3. Flow cytometry size-gated synaptosome counting in temporal cortex. a, Representative flow cytometer plots of pooled synaptosomes from the TCx of 6 CTRL, 6 MCI and 6 AD subjects (each point is a technical replicated of the pool). Synaptosome particles were labeled with anti-PSD95 (1:80, Novus-NB300-556AF647) or Anti-Gephyrin (1:100, Abcam-Ab32206) and size gated using side scatter/forward scatter. **b,** Excitatory synaptosomes positive for PSD95 marker were significantly reduced in MCI and AD compared to CTRL (one-way ANOVA, F (2, 6) = 10.03, p = 0.01 followed by Dunnett multiple comparison test, MCI, p = 0.01, and AD, p = 0.02). **c,** Number inhibitory synaptosomes positive for gephyrin did not change among diagnosis groups (one-way ANOVA F (2, 6) = 2.32, p = 0.18). **d,** Overall, we observed a significant reduction in total number of synaptosomes of MCI and AD compared to the CTRL (one-way ANOVA F (2, 6) = 15.04, p = 0.005 followed by Dunnett's multiple comparisons test CTRL vs MCI, p = 0.006; CTRL vs AD p = 0.005). Mean values of particles positive for PSD95^{+} per microliter for CTRL, 474959; MCI, 398543; AD, 406154. For gephyrin^{+}, CTRL, 393584; MCI, 370346; AD, 361304. Total number of particles is the sum of PSD95^{+} plus gephyrin^{+}: CTRL, 868543; MCI, 768889; AD, 767458. Whiskers represent standard error.

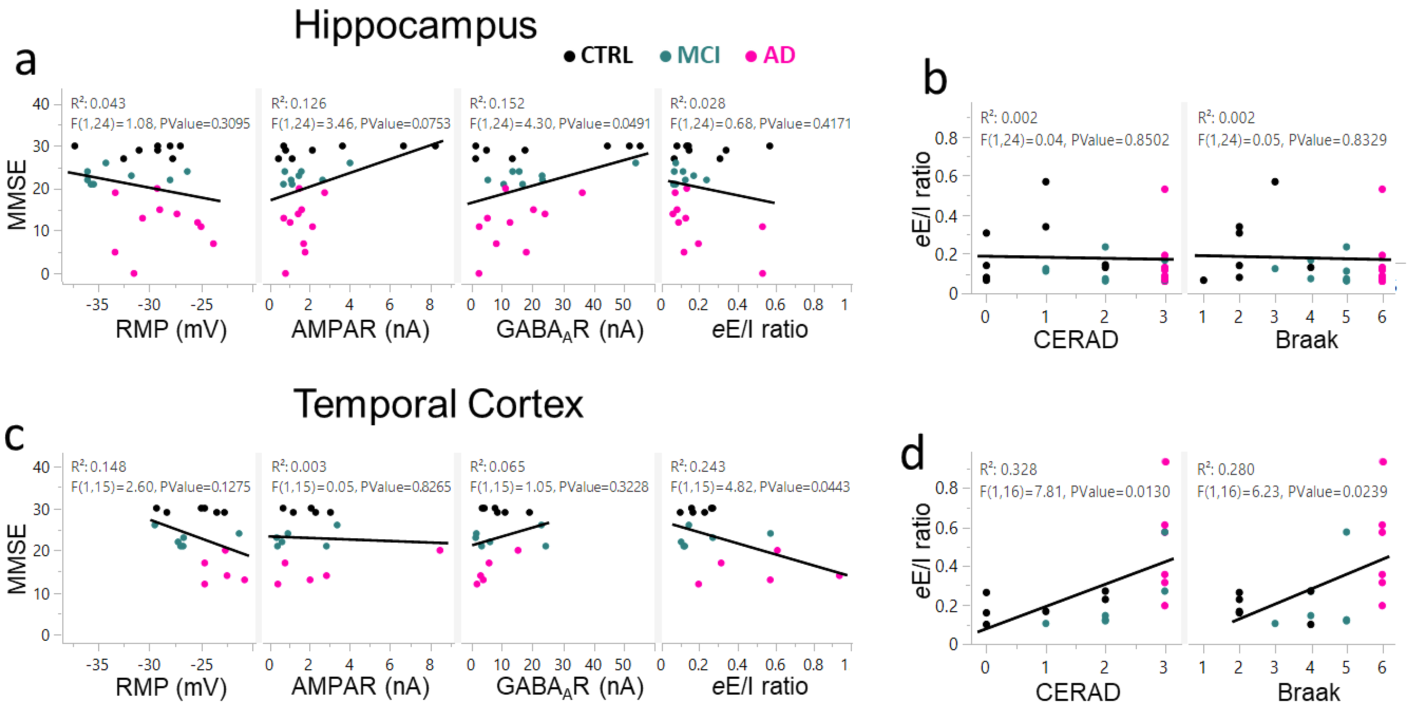

Supplementary Figure 4. Relationship between electrophysiological parameters and MMSE scores. Linear correlations between the resting membrane potential (RPM), the maximum amplitude of responses elicited by microtransplanted AMPA receptors (AMPARs), GABA receptors ($GABA_A$Rs), and their ratio (*e*E/I) with cognitive performance score in the hippocampus (**a**) and the temporal cortex (**c**). AMPARs were activated with 100 µM kainate and $GABA_A$Rs with 1 mM GABA. Resting membrane potential of microtransplanted oocytes do not correlate with MMSE in temporal cortex and hippocampus. In hippocampus, the higher the amplitude of $GABA_A$Rs current the better the cognitive performance of the subject (MMSE). A similar trend was observed for AMPARs currents. In the temporal cortex neither $GABA_A$R nor AMPAR currents correlated with the MMSE. Whereas the eE/I ratio had no correlation with MMSE in the hippocampus, there was an association between the *e*E/I ratio and MMSE in the temporal cortex. *e*E/I ratio correlated with plaques and Braak stage in the temporal cortex (**d**) but not in hippocampus (**b**).

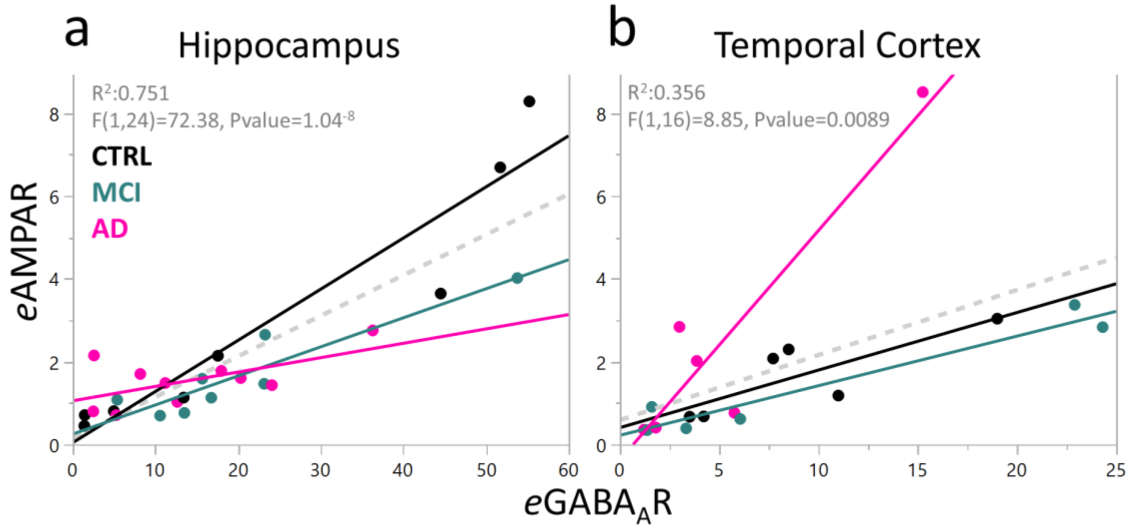

Supplementary Figure 5. Strong correlation between synaptic excitatory and inhibitory currents in hippocampus and temporal cortex. Microtransplanted oocytes with hippocampus (**a**) or temporal cortex (**b**) synaptosome fractions from CTRL (black), MCI (aqua) and AD (magenta) subjects, were tested with GABA 1mM and kainate 100µM eliciting $GABA_A$Rs and AMPARs currents, respectively. Correlation values in the figure represent the linear to all data (dotted line). The linear fits to the data are color coded as per the insert. Correlation values for hippocampus were $R^2 = 0.899$, $p = 0.003$; $R^2 = 0.845$, $p = 0.0012$ and $R^2 = 0.36$, $p = 0.06$ for control, MCI and AD, respectively. For the TCx the values for these groups were $R^2 = 0.66$, $p = 0.049$; $R^2 = 0.92$, $p = 0.0021$ and $R^2 = 0.86$, $p = 0.008$.

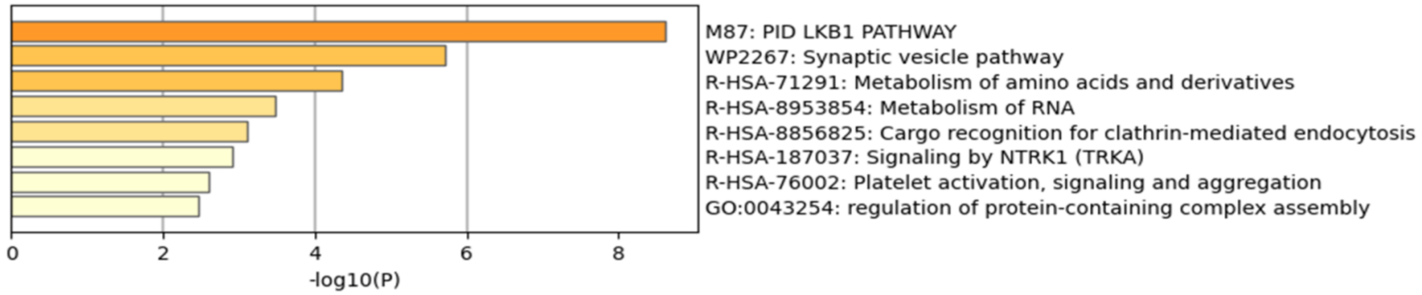

Supplementary Figure 6. Metascape analysis of proteins differentially expressed across groups. Clusters of the gene ontology (GO) analysis for cellular component of proteins that were either reduced or increase in MCI, AD respect to controls with an FDR $p < 0.05$. For the analysis, we used background geneset "brain" available on https://www.syngoportal.org/.

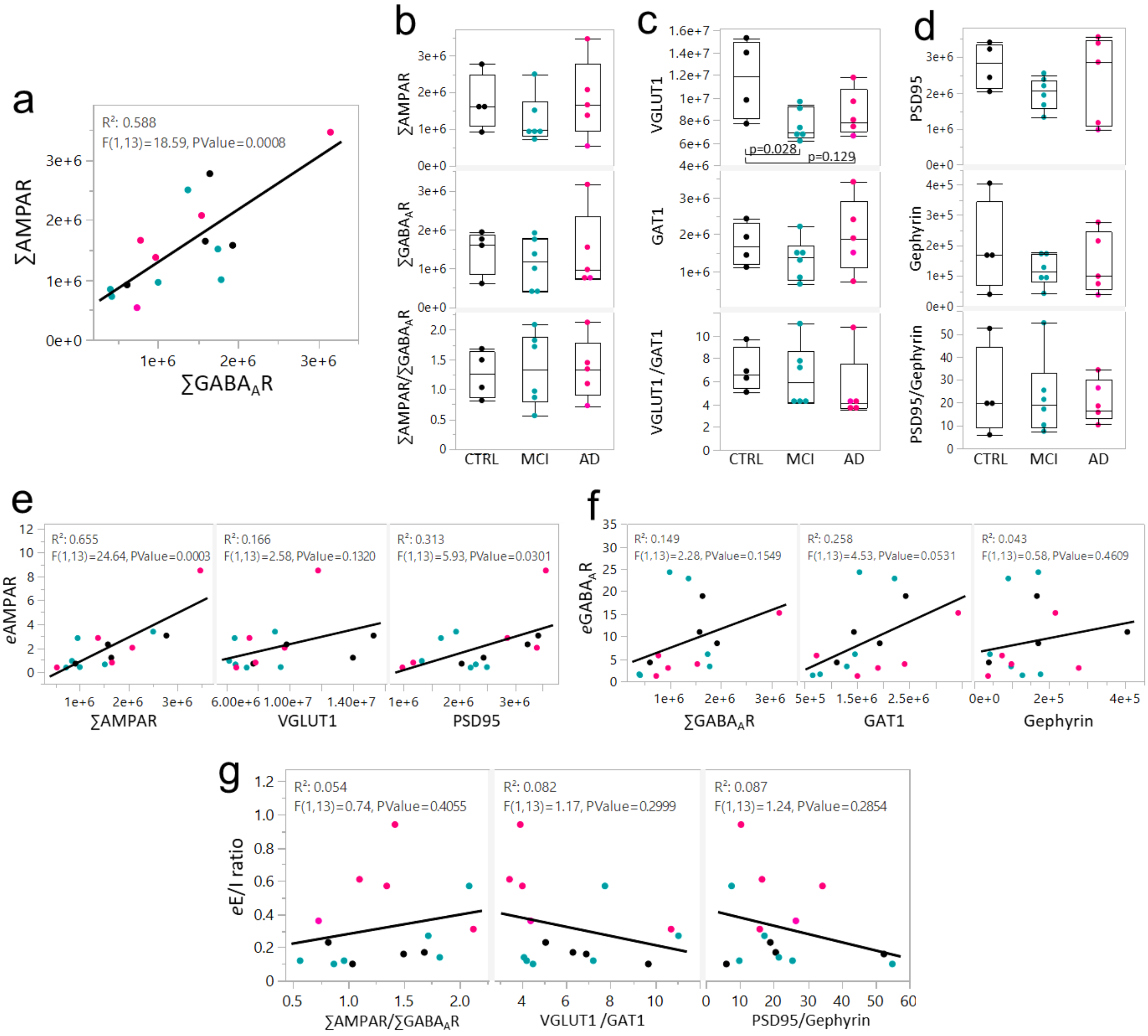

Supplementary Figure 7. Proteomic analysis of excitatory and inhibitory synaptic receptors and markers in TCx. a, Sum of all AMPAR subunits (ΣAMPARs) and sum of all $GABA_A$R subunits (Σ$GABA_A$Rs) showed significant correlation (Pearson's correlation). **b**, No differences in receptors abundance (one-way ANOVA, ΣAMPARs $F(2, 14) = 0.70$, $p = 0.52$; Σ$GABA_A$R $F(2, 14) = 0.31$, $p = 0.74$), or their ratio ($F(2, 14) = 0.03$, $p = 0.97$), was found across groups. **c,** We observed a decrease of the glutamate transporter, VGLUT1, in MCI compared to CTRL, and a similar, but not significant trend in AD (One-way ANOVA, $F(2, 14) = 3.98$, $p = 0.047$ followed by multiple comparison Dunnett's test CTRL vs MCI, $p = 0.028$, CTRL vs AD, $p = 0.129$). No differences were observed for the synaptic GABA transporters GAT1 (One-way ANOVA, $F(2, 14) = 1.10$, $p = 0.36$) and the ratio VGLUT1/GAT1 ($F(2, 14) = 0.48$, $p = 0.62$). **d,** No differences within diagnosis were found for levels of synaptic scaffolds PSD95 and gephyrin (One-way ANOVA, PSD95 $F(2, 14) = 1.10$, $p = 0.36$; gephyrin $F(2, 14) = 0.71$, $p = 0.51$) and the proteomic ratio (*p*E/I ratio) defined as PSD95/GPHN ($F(2, 14) = 0.06$, $p = 0.94$). **e**, Electrophysiological amplitude of AMPARs currents highly correlated with abundance of AMPAR subunits (Pearson's correlation), and PSD95, but not with VGLUT1 **f**, Electrophysiological GABA currents did not show correlation with any of the inhibitory proteomic marker: abundance of $GABA_A$R subunits, gephyrin levels, and GAT1. **g,** The *e*E/I ratio did not correlate with the *p*E/I, VGLUT1/GAT1, and ΣAMPARs/Σ$GABA_A$Rs ratios.

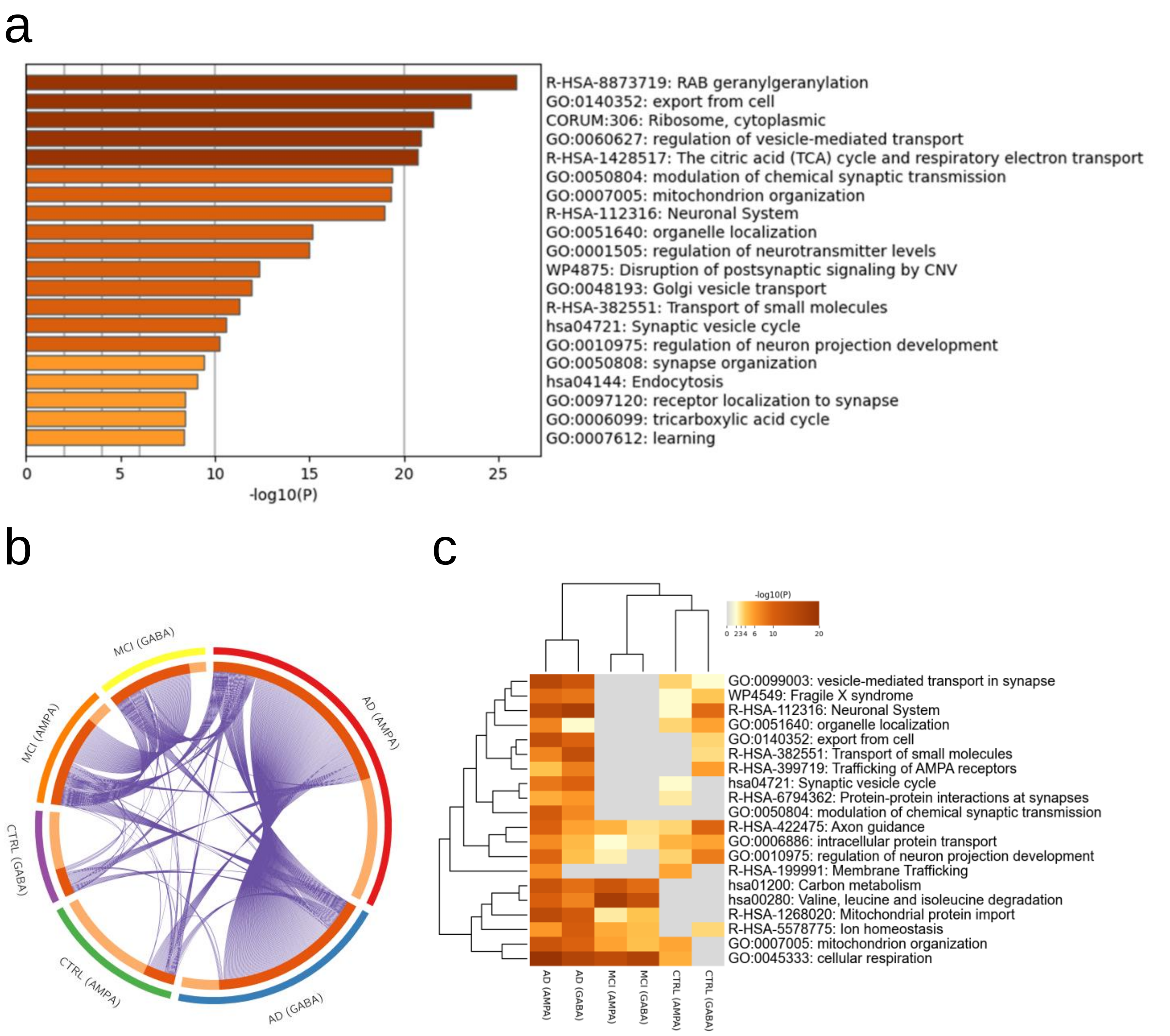

Supplementary Figure 8. Metascape analysis of proteins positively correlated with electrophysiological responses of AMPAR and $GABA_AR$. a, Clusters of the gene ontology (GO) analysis for cellular component of proteins positively correlated with electrophysiological responses of AMPAR and $GABA_AR$, implemented in Metascape for all the cohort combined. For the analysis, we used background geneset "brain" available on https://www.syngoportal.org/. **b**, Circos plot showing how the proteins that correlate with AMPARs and $GABA_ARs$ responses of each group overlap. On the outside, each arc represents the identity of each gene list. On the inside, each arc represents a gene list, where each gene member of that list is assigned a spot on the arc. Dark orange color represents the genes that are shared by multiple lists and light orange color represents genes that are unique to that gene list. Purple lines link the same gene that are shared by multiple gene lists (notice a gene that appears in two gene lists will be mapped once onto each gene list, therefore, the two positions are purple linked). The greater the number of purple links and the longer the dark orange arcs imply greater overlap among the input gene lists. **c**, Clusters of GO enrichment for the proteins that correlate with electrophysiological responses per group.

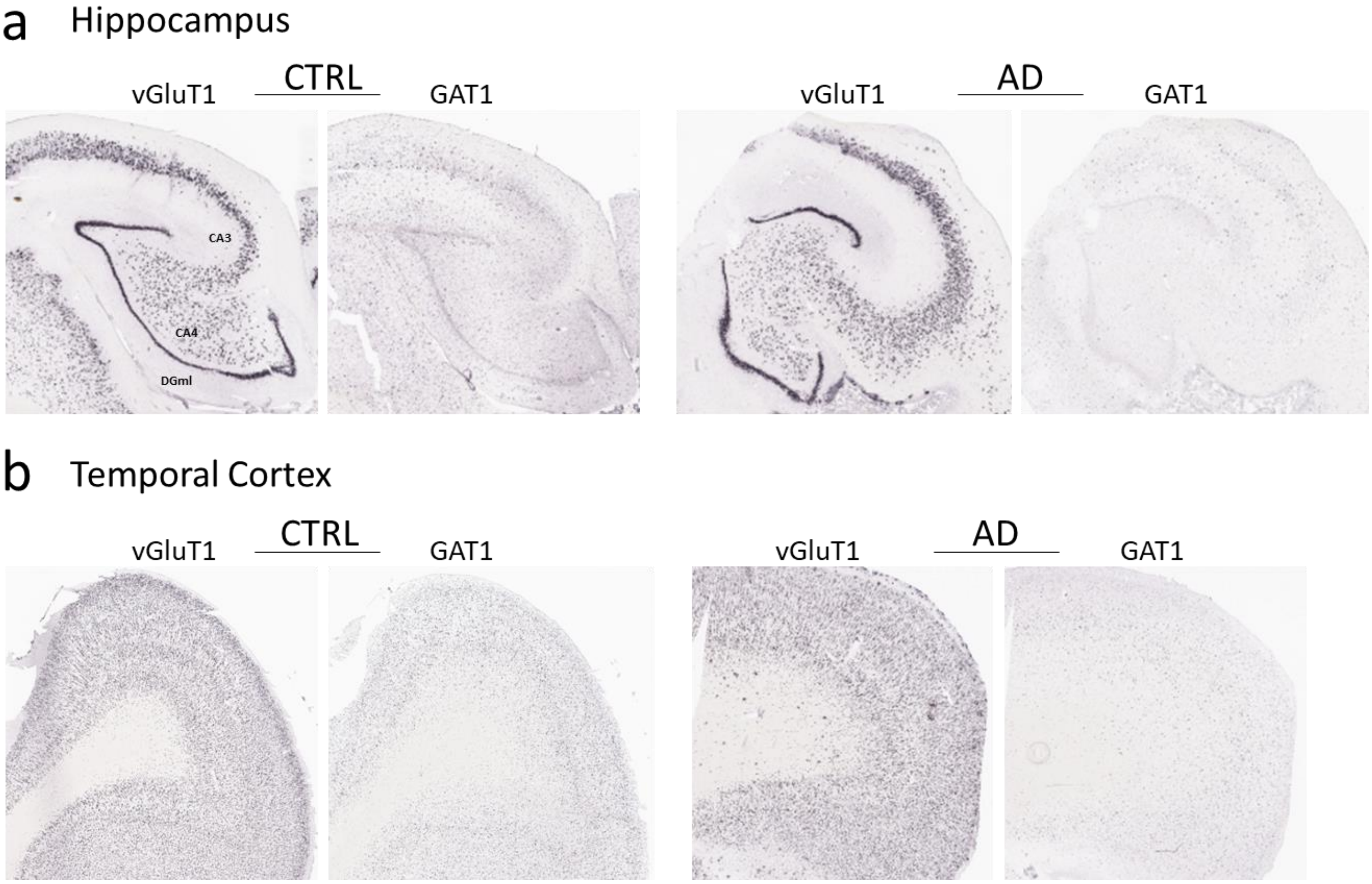

Supplementary Figure 9. Representative low power *in situ* hybridization for vGluT1 and GAT1 mRNAs. Hippocampus **a**, and temporal cortex **b**, from CTRL and AD cases. For hippocampus, the subfields used for quantification are shown: CA3, CA4 and the dentate gyrus molecular layer (DGml).

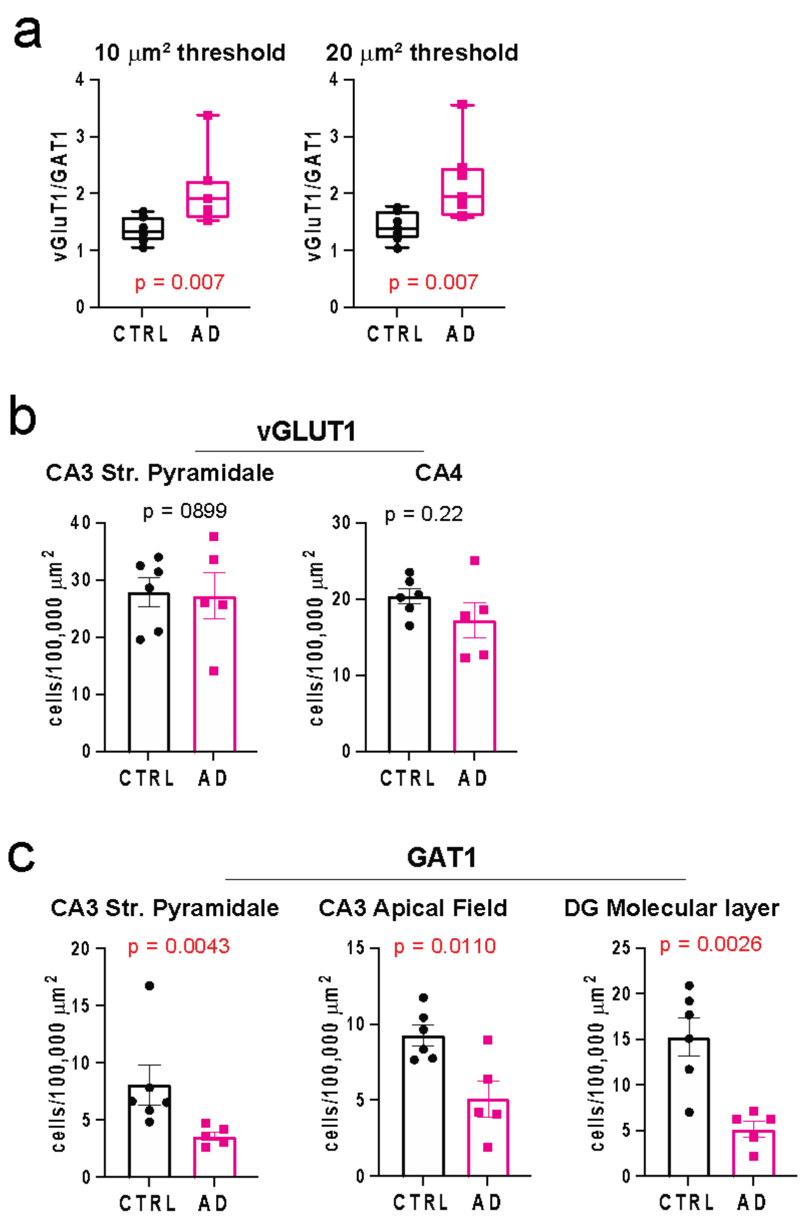

Supplementary Figure 10. In Situ hybridization analysis using different size thresholds. a, Box plots show the ratio of vGluT1$^+$ to GAT1$^+$ mRNA labeled cells in temporal cortex analyses using 10 and 20 µm^2 minimum thresholds; plots show median values, 25th and 75th percentiles, and minimum and maximum range. Like the 30 µm^2 threshold used for final analyses (Figure 5), similar results were obtained using the 10 and 20 µm^2 thresholds with the AD group having a significantly higher E/I ratio in all analyses (***p* = 0.007, two-tailed unpaired Mann Whitney test for both). **b,** Quantification of vGluT1$^+$mRNA cells in CA3 stratum pyramidale and CA4. Numbers of labeled cells were not statistically different between AD and Control groups (*p* = 0.8999 for CA3 and *p* = 0.2191 for CA4, two-tailed unpaired Student's t-test). **c,** Quantification of GAT1$^+$mRNA cells in CA3 stratum pyramidale, CA3 apical dendritic field, and dentate gyrus (DG) molecular layer. For all three fields, the AD group had significantly less inhibitory neurons than the controls (***p* = 0.0043 for CA3 stratum pyramidale, two-tailed unpaired Mann Whitney test; **p* = 0.0110 for CA3 apical dendritic field, two-tailed unpaired Student's *t*-test; and ***p* = 0.0026 for DG molecular layer, two-tailed unpaired Student's *t*-test). All error bars represent standard errors.
